# Rituximab in Multiple Sclerosis: Are We Ready for Regulatory Approval?

**DOI:** 10.3389/fimmu.2021.661882

**Published:** 2021-07-06

**Authors:** Serena Brancati, Lucia Gozzo, Laura Longo, Daniela Cristina Vitale, Filippo Drago

**Affiliations:** ^1^ Clinical Pharmacology Unit/Regional Pharmacovigilance Centre, University Hospital of Catania, Catania, Italy; ^2^ Department of Biomedical and Biotechnological Sciences, University of Catania, Catania, Italy; ^3^ Centre for Research and Consultancy in HTA and Drug Regulatory Affairs (CERD), University of Catania, Catania, Italy

**Keywords:** multiple sclerosis, rituximab, off-label, regulatory issue, disease-modifying drugs

## Abstract

Despite the availability of a lot of effective disease-modifying drugs, multiple sclerosis (MS) (in particular the progressive forms) still represents an important unmet medical need, because of issues in terms of effectiveness, duration of response, safety, and patient compliance. An increasing body of evidence from randomized clinical trials and real-world data suggest that rituximab is a highly effective alternative in both relapsing and progressive MS, with a low discontinuation rate, related to a good benefit/risk profile, and a good compliance. To date, the use of rituximab in patients with multiple sclerosis is not in accordance with the authorized product information (off-label use). However, the use of this medicine is widespread in several countries, and in some cases, it is the most commonly used disease-modifying drug for MS subtypes. This use could be officially recognized by national regulatory authorities, according to specific procedures, to ensure equal access for patients to a safe and effective option.

## Introduction

Multiple sclerosis (MS) is the most common chronic demyelinating disorder of the central nervous system (CNS), affecting more than 2.8 million people worldwide in 2020, with a global median prevalence of 36 cases per 100,000 people, and an average incidence rate of 2.1 per 100,000 people per year (1, 2). MS primarily affects young adults, with the age of onset between 20 and 40 years, and it could be considered the second-most expensive chronic condition behind congestive heart failure in the US (3). The clinical manifestations and course of MS are heterogeneous, with different degrees of severity, from an initial clinically isolated syndrome (CIS), to a relapsing–remitting form (RRMS) and the progressive development of permanent neurological deficits and disability (known as secondary progressive MS, SPMS). Moreover, some patients have a progressive disease from the onset, known as primary progressive form (PPMS) (4). CIS and RRMS are typically characterized by active white matter demyelinating lesions, with heavy immunological infiltration and activation (5), whereas the progressive forms are mainly characterized by inactive lesions, reduced inflammation and neurodegeneration (6, 7).

The physiopathological mechanisms behind the damage are still incompletely understood (8). T cells appear early in lesion formation, and the disease is considered to be autoimmune, initiated by autoreactive lymphocytes that mount aberrant responses against CNS autoantigens, the precise nature of which, however, have not been routinely identified (9, 10). B cells and their plasma cell derivatives also produce antibodies, including clonally expanded immunoglobulin G (IgG) oligoclonal bands (OCBs) detectable in the cerebrospinal fluid of most patients with MS (11). However, B cells probably contribute mainly through antibody-independent mechanisms, due to an abnormal cytokine response profile — with a propensity to produce pro-inflammatory cytokines (including IL-6, GM-CSF, TNF, and lymphotoxin-α) — that can induce aberrant Th1 cell and Th17 cell responses and pro-inflammatory myeloid cell responses, which could in turn contribute to the cellular immune cascades involved in first phases of the pathology and in relapses (12–14). Treg cells can be responsible in inducing remission in MS, through the downregulation of immune responses (15), and activated pro-inflammatory cells may be more likely to be killed by other immune cells (16). In later stages of the disease, ongoing inflammation in the CNS might contribute to the propagation of tissue injury, in terms of neuro-axonal degeneration, astrocyte, and oligodendrocyte damage, and to the clinical manifestations of progressive disease (7). The different inflammatory characteristics among progressive forms and RR forms of MS may explain the lack of efficacy of most disease modifying therapies (DMTs), which are typically systemic anti-inflammatory drugs.

Cognitive impairment (impairment in information processing speed, episodic memory, attention, efficiency of information processing, and executive function), which can start in the earliest phases of the disease but is more frequent and more pronounced in chronic progressive MS, worsens over time and affects the patient’s daily life activities (17).

Optimal MS management requires coordinated and comprehensive care from health care professionals with expertise in the complexities of MS (18, 19). Untreated relapses and progression of disease restrict participation in usual activities and increase the risk for serious morbidity. The ultimate goal of modern MS therapies is to achieve no evidence of disease activity (NEDA) in which the therapy has halted relapses and disability progression, as well as new and active magnetic resonance imaging (MRI) lesion development. The treatment of MS includes DMTs, which are used to reduce inflammatory disease activity and its long-term clinical consequences; the treatments for the management of MS relapses and symptomatic treatments are used for short-term amelioration of MS symptoms, such as impaired walking capability, spasticity, pain, loss of bladder and bowel control, and neuropsychiatric symptoms (4).

The most established treatment for the acute management of MS relapses is high-dose corticosteroids. In particular, current protocols typically include 3 to 5 days of intravenous methylprednisolone (20). Relapses that do not respond to corticosteroids can be treated with plasma exchange (3–5 courses) or intravenous immunoglobulins.

DMTs effectively reduce the inflammatory activity, relapse rate, and disability progression, although safety concerns, individual immunological changes, and issues with compliance make their long-term use challenging. To date, several DMTs, with different routes and frequencies of administration, mechanisms of action, effectiveness, and safety profiles, have been approved for the treatment of RRMS in EU — including subcutaneous interferon-β (IFNβ)-1a, IFNβ-1b, and pegIFNβ-1a, subcutaneous glatiramer acetate, small-molecule oral agents (cladribine, dimethyl fumarate, fingolimod, ozanimod, teriflunomide), intravenous monoclonal antibodies (mAbs) (alemtuzumab, natalizumab, ocrelizumab), and intravenous mitoxantrone — offering to physicians the possibility of tailoring therapy to individual patient needs (**Table 1**). Effective treatments for the progressive forms of MS are more limited, with only a small number of therapeutic agents available with beneficial effects.

**Table 1:** Disease-modifying therapies currently licensed for the treatment of MS; 1a) Common first-line treatment; 1b) Common second-line treatments.

A							
DMT	Administration route, dosage and posology	Mechanism of action	Efficacy	Main adverse effects/Safety issues	Monitoring requirements	First EMA approval (year)	Indication
INFβ-1b	Subcutaneous injection, 250 mcg every other day	Not fully understood. Autocrine and paracrine actions *via* activation of the IFN receptor on leucocytes (21)	Moderate	Injection site reactions, flu-like symptoms, abnormal LFTs, lymphopenia, leukopenia, depression (and suicidal ideation), thyroid dysfunction, neutralizing antibodies	At baseline and periodically during treatment: full blood count, differential leukocyte count, platelet count, liver function tests, and TFTs.	1995	CIS
							RMS
INFβ-1a	Intramuscular injection 30 mcg once a week or subcutaneous injection; 22 mcg or 44 mcg three times a week	The same as above	Moderate	The same as above	The same as above	1997	CIS
							RMS
Peg-INFβ-1a	Subcutaneous injection, 125 mcg once every 2 weeks	The same as above	Moderate	The same as above	The same as above	2014	RRMS
Glatiramer acetate	Subcutaneous injection, 20 mg daily or 40 mg three times per week	Unclear. Immuno-modulatory and neuroprotective effect through various mechanisms. MBP mimetic, thus competes with MBP antigens to bind with MHC II (22).	Moderate	Injection site reactions, post-injection reactions (vasodilatation, rash, dyspnea, chest pain within minutes), mood disturbance, hypersensitivity reaction, cutaneous necrosis	None required	2005	CIS
							RRMS
Dimethyl fumarate	Oral capsule, 240 mg twice a day	Not fully understood. Activates the Nrf2 pathway to protect against oxidative stress–induced cellular injury and loss in neurons and astrocytes (23)	Moderate/High	Flushing, gastrointestinal symptoms (abdominal pain, diarrhea, and nausea), pruritus/rash, anaphylactic reactions, lymphopenia, infections (VZ), PML, abnormal LFTs, proteinuria	At baseline and periodically during treatment: full blood count, differential leukocyte count, LFTs, renal function monitoring	2014	RRMS
Teriflunomide	Oral tablets, 14 and 7 mg daily	Inhibits proliferation of activated T and B lymphocytes *via* mitochondrial dihydroorotate dehydrogenase inhibition (24)	Moderate	Hair thinning, gastrointestinal symptoms (nausea, diarrhea), abnormal LFTs, impaired bone marrow function with anemia, leukopenia, neutropenia, thrombocytopenia, infections, peripheral neuropathy, skin AEs, increased blood pressure, respiratory effects (interstitial lung disease), pancreatitis, teratogenicity	At baseline and periodically during treatment: blood pressure, LFTs (fortnightly for 6 months then every 8 weeks), full blood count	2013	RRMS

B							
DMT	Administration route, dosage and posology	Mechanism of action	Efficacy	Main adverse effects/Safety issues	Monitoring requirements	First EMA approval (year)	Indication
Fingolimod	Oral capsule, 0.5 mg daily (0.25 mg daily for pediatric patients ≤ 40 kg)	S1P agonist -prevents egress of lymphocytes from lymph nodes (25, 26)	High	Headache, diarrhea, back pain, elevated liver enzymes, bradyarrhythmia, and/or atrio-ventricular block (first dose), hypertension, respiratory effects, lymphopenia, infections (VZ), PML, macular edema, increased risk of malignancies (basal cell carcinoma), hepatic injury, teratogenicity	First-dose observation protocol (6-h monitoring of heart rate and blood pressure). Baseline: full blood count, serum Ig levels, serology (VZV, HIV 1 and 2, hepatitis B and C, syphilis), LFTs, skin examination. Periodically during treatment: full blood count, blood pressure, ECG, skin examination, ocular examination at 3 months	2011	Highly active RRMS* (adults and pediatrics from 10 years)
Natalizumab	Intravenous infusion, 300 mg every 4 weeks	Selective inhibitor of VLA-4 (α4β1) integrins, preventing leukocyte migration across BBB (27)	Very high	Arthralgia, urticaria, infusion reactions, opportunistic infections (VZ, encephalitis, meningitis, PML), hepatic injury	Baseline and periodically during treatment: full blood count, LFTs, JCV serology and MR, neutralizing antibodies	2006	Highly active RRMS* (adults)
							Adolescents (12-18 years) with severe and rapidly evolving RRMS* not eligible to fingolimod (648/1996 law)
Alemtuzumab	Intravenous infusion, 12 mg, first course: daily for 5 days; second course: daily for 3 days, 1 year after the first course	Anti-CD52 mAb depleting B cells, T cells, monocytes, macrophages, and dendritic cells (immune reconstitution therapy) (28, 29)	Very high	Infusion reactions, profound lymphopenia, infections (herpes simplex and zoster), secondary autoimmunity (as thyroid disorders, immune thrombocytopenia, purpura, glomerular nephropathies), Hemophagocytic lymphohistiocytosis (HLH), serious cardiovascular disorders	Baseline: full blood count, urine analysis, LFTs, TFTs, serum immunoglobulin levels, serology (VZV, HIV 1 and 2, hepatitis B and C, syphilis), TB elispot, cervical smear (HPV). Follow-up (for 48 months after last course): monthly full blood count, urine analysis and 3-monthly TFTs	2013	Highly active RRMS* (adults)
Cladribine	Oral 10 mg tablets, cumulative dose of 3.5 mg/kg over 2 years, administered as 1.75 mg/kg treatment cycle per year. Tablets given for 4–5 days in months 1 and 2 in year 1 and the cycle is repeated in year 2 (8–10 days of treatment per year)	Deoxyadenosine (purine) analog, adenosine deaminase inhibitor, selective T- and B-cell depletion (immune reconstitution therapy) (28, 30)	High	Severe lymphopenia, infections (VZ), TB/LTB reactivation, increased risk of malignancies, teratogenicity	Baseline: full blood count (before each treatment year), LFTs, TFTs, serum immunoglobulin levels, serology (VZV, HIV 1 and 2, hepatitis B and C, syphilis), TB elispot, pregnancy test, and cervical smear. Follow-up: full blood count 2 and 6 months after start of treatment in each treatment year	2017	Highly active RMS (including RRMS and SPMS)
Ocrelizumab	Intravenous infusion, 600 mg twice a year (initially 300 mg/250 ml IV, followed 2 weeks later by second dose of 300 mg/250 ml IV; subsequent dosing 600 mg/500 ml IV 6 monthly)	Anti-CD20 mAb, B-cell depleter (immune reconstitution therapy) (28, 31)	Very high	Infusion reactions, infections, PML, increased risk of malignancy, possible hypogammaglobinemia with prolonged use	Baseline: full blood count, LFTs, TFTs, serum immunoglobulin levels, serology (VZV, HIV 1 and 2, hepatitis B and C, syphilis), TB elispot, cervical smear. Follow-up: annual serum immunoglobulin levels	2018	RMS
							PPMS
Mitoxantrone	Intravenous infusion, 12 mg/m^2^ every 3 months or 5 mg/m^2^ every 3 months	Immune deplete (topoisomerase II inhibitor) (32)	Very high	Leukopenia, hair loss, nausea, vomiting, infections, cardiomyopathy (congestive heart failure), amenorrhea, myelosuppression, secondary acute myeloid leukemia, myelodysplastic syndrome, infections, renal failure, teratogenicity	Baseline: full blood count, LFTs, TFTs, serum immunoglobulin levels, serology (VZV, HIV 1 and 2, hepatitis B and C, syphilis), TB elispot. Follow-up: 3-monthly (predosing) full blood count	2015**	Highly active RMS associated with rapidly evolving disability (patients not eligible to other therapeutic alternatives)
Siponimod	Oral tablets, 2 mg daily (maintenance dose after 5 days titration)	S1P agonist (33)	Very high	Lymphopenia, infections (including cryptococcal and herpes viral infections), macular edema, bradyarrhythmia atrioventricular conduction delays, hypertension, respiratory effects, liver injury, hypertension, skin malignancies, fetal risk.	First dose monitoring for patients with sinus bradycardia, first- or second-degree atrio-ventricular block or a history of myocardial infarction or heart failure. Baseline: CYP2C9 genotyping; vital signs and ECG; full blood count; serology (VZV, HIV 1 and 2); ocular examination; LFTs. Follow-up: full blood count; ocular examination at 3 months; skin examination; LFTs; neurologic and psychiatric examination	Jan 2020	SPMS
Ozanimod	Oral capsules, 0,92 mg daily (maintenance dose after 7 days titration)	S1P agonist (34)	Very high	Bradycardia, hypertension, LFTs alterations, liver injury, infections (PML risk), increased risk of malignancies (skin malignancies), macular edema, PRES, respiratory effects, fetal risk	First dose monitoring for patients with sinus bradycardia, first or second degree AV block or a history of myocardial or heart failure. Baseline: full blood count, blood pressure, ECG, LFTs, ocular examination. Follow-up: full blood count, LFTs, blood pressure, ocular examination	May 2020	RMS

BBB, blood-brain barrier; CIS, clinically isolated syndrome; INF, interferon; MBP, myelin basic protein; MHC II, class II major histocompatibility complex; MS, multiple sclerosis; NGF, nerve growth factor; Nrf2, nuclear factor erythroid 2-related factor 2; PPMS, primary progressive multiple sclerosis; RMS, relapsing multiple sclerosis; RRMS, relapsing remitting multiple sclerosis; SPMS, secondary progressive multiple sclerosis; LFTs, liver function tests; PML, progressive multifocal leukoencephalopathy; PRES, posterior reversible encephalopathy syndrome; S1P, sphingosine 1-phosphate; JCV, John Cunningham virus; TB, tuberculosis; TFTs, thyroid function tests; VZ, varicella zoster.

*Highly active disease despite a full and adequate course of treatment with at least 1 disease modifying therapy OR 2+ disabling relapses in previous year and with MRI activity including enlarging T2 lesions. **Mitoxantrone has been first authorized in 2000 as antineoplastic.

Because of the wide variability in the disease course and in the individual responses to treatment, access to several DMTs, with different routes of administration and dosing schedules, mechanisms of action, efficacy and safety profiles, contraindications, and side effects, is essential to ensure a good long-term control of the disease.

Escalation therapy is appropriate for most patients with non-aggressive RRMS, provided that they are closely monitored to detect suboptimal response or disease progression. Subjects with an intolerable degree of disease activity despite high-efficacy treatments may be treated with alternative immunosuppressive agents, such as mitoxantrone (currently authorized for the treatment of highly active relapsing MS associated with rapidly evolving disability in the absence of other therapeutic alternatives), cyclophosphamide, and azathioprine (35, 36).

In patients with active SPMS, current ECTRIMS/EAN guidelines recommend (weak recommendation) the following: IFNβ-1a or -1b, taking into account the efficacy, safety, and tolerability profiles of these drugs; mitoxantrone, taking into account its efficacy and specifically considering the safety and tolerability of the drug (cardiotoxicity, delayed congestive heart failure, myelosuppression, and acute treatment–related leukemia); and ocrelizumab or cladribine (35). EU approval of siponimod is too recent for its consideration in these guidelines. Another anti-CD20 agent, ofatumumab, that can be self-administered once monthly at home subcutaneously, has been approved in August 2020 by FDA for the treatment of relapsing form of MS, including CIS, RRMS, and active secondary progressive disease, with an expected approval in Europe by the first half of 2021.

For patients with PPMS, ocrelizumab represents, to date, the only authorized treatment.

### The Role of Anti-CD20 in MS

The reduction of B-cells demonstrated to be an effective therapeutic approach for the progression of CNS autoimmune diseases (37).

There are three major mAbs targeting CD20+ B-cells, rituximab, ocrelizumab, and ofatumumab. The mechanisms of apoptotic B-cell depletion include antibody-dependent cell-mediated phagocytosis, antibody-dependent cellular cytotoxicity (ADCC), and complement-dependent cytotoxicity (CDC) (28, 31). Recent studies have shown also a depleting action on CD20+ T cells, which are shown to be present in MS patients, suggesting an alternative contributing mechanism (38).

Rituximab is the first anti-CD20 therapy to be used in MS. It is a chimeric antibody, approved since 1997 for hematological and autoimmune disorders. However, it is not approved for use in MS, but is commonly prescribed as off-label treatment.

On the contrary, its humanized surrogate ocrelizumab received EMA and FDA approval for the treatment of patients with relapsing forms of MS or with early PPMS. In two phase III trials, OPERA I and OPERA II, ocrelizumab reduced annual relapse rate (ARR) up to 47% and disability progression by 40% compared with subcutaneous IFNβ-1a (39). Ocrelizumab also induced a reduction in the count of T1 gadolinium (GAD)-enhancing lesions (up to 94%) and the mean number of new or newly expanding lesions on T2-weighted MRI imaging. 47.9% and 47.5% of patients treated with ocrelizumab in OPERA I and OPERA II, respectively, after 96 weeks demonstrated no evidence of relapses, disability progression, and T2- or GAD-enhancing T1 lesions, without new safety concerns (40). In addition to the robust phase III data in RRMS, ocrelizumab had also favorable phase III data in PPMS (41). In the ORATORIO trial, patients receiving ocrelizumab had lower disability progression at 3 and 6 months and showed a reduced volume of T2 hyperintense lesions and a significant improvement in brain volume loss compared with placebo. Long-term follow-up data from the open-label extension of ORATORIO trial showed persistent efficacy in patients treated continuously with ocrelizumab up to 6.5 study years, with no evidence for increasing risk of adverse events (AEs) related to cumulative exposure (42). The only concern was a decrease in serum immunoglobulin concentration below the lower limit of normal, where the clinical significance is not clear (43). The most common AEs associated with the use of ocrelizumab are infections followed by infusion-related reactions (IRRs) (44). One observational study reported a higher risk of AE-related discontinuations for ocrelizumab versus rituximab (rate ratio [RR], 2.66; 95% confidence interval [CI], 1.09-6.47) (45). Current recommendations to reduce the risk of an IRR include pre-medication with intravenous methylprednisolone and an antihistamine and monitoring of patients during and after the infusion (46). Interestingly, a shorter infusion period (2 h *versus* 3.5 h) was not associated with an increased risk of IRRs (47), and EMA has recently authorized the 2-h infusion time for second and subsequent doses. The most commonly reported serious AEs (SAEs) are serious infections, followed by neoplasms. Treatment with B-cell-depleting anti-CD20 frequently results in a decrease in total immunoglobulins (IgG, IgM, IgA), typically associated to the occurrence of recurrent or complicated serious infections (45, 46, 48–51). As of December 2020, 10 cases of progressive multifocal leukoencephalopathy (PML) (nine cases had prior exposure to either natalizumab or fingolimod, and one case had no prior exposure (52), and six other serious opportunistic infections (including systemic Pasteurella infection, multisegmental herpes zoster infection, enterovirus-induced fulminant hepatitis requiring a liver transplant, Candida sepsis, viral meningitis) have been reported (44). However, because of its relatively recent marketing authorization, PML risk in patients treated with ocrelizumab has not yet been well established. Overall, 64 cases of neoplasms have been reported among patients treated with ocrelizumab across all the trials, to which eight cases reported in observational studies and a total of 95 cases of breast cancer reported among women exposed outside of clinical trials have been added (44). A much longer follow-up in large populations treated in a real-world setting is necessary to assess the real correlation between malignancies and ocrelizumab treatment. Finally, cases of neutropenia have been described after ocrelizumab treatment, as well as one case of a drug-induced hypersensitivity syndrome (DRESS).

### Clinical Data Supporting the Use of Rituximab in Multiple Sclerosis

Rituximab recognizes a similar epitope of CD20 protein to that ocrelizumab, but with a relatively higher binding affinity (53). As ocrelizumab, rituximab induces cell death through apoptosis, ADCC, antibody-dependent cell-mediated phagocytosis, and CDC. Because of the differences in the Fc regions, rituximab induces more CDC and less ADCC than ocrelizumab, being, accordingly, theoretically more prone to induce infusion-related side effects (53, 54). As other monoclonal antibodies, rituximab does not pass readily across the BBB, and its CSF concentration has been estimated to reach only 0.1% of that in serum after intravenous administration (55); nevertheless, a profound depletion of intrathecal B cells with standard intravenous doses is evident (56).

It was initially approved for CD20+ non-Hodgkin lymphoma and subsequently for CD20+ chronic lymphocytic leukemia, rheumatoid, granulomatosis with polyangiitis and microscopic polyangiitis, and pemphigus vulgaris (48).

Available relevant literature (updated on March 2021) was searched on MEDLINE (PubMed), applying the medical subject headings (MeSH) terms “multiple sclerosis” and “rituximab” and “efficacy” and “safety.”

We selected peer-reviewed, full-text, and English language manuscripts, randomized-controlled trials (RCTs), prospective studies, non-randomized clinical trials, retrospective studies, and studies made from registries. We excluded meta-analyses and reviews.

Clinical trials and real-world data supporting the use of rituximab in patients with MS are reported below and summarized in **Supplementary Table 1**.

### Clinical Trials

The first trial with rituximab in MS was an open-label phase I study providing an initial assessment of safety, tolerability, and activity of the drug in a small cohort of 26 patients with active RRMS, aged 18 to 55 years and mostly not treatment naïve, followed for 72 weeks (57). Patients received intravenous rituximab 1,000 mg on days 1 and 15, and a second course of treatment on weeks 24 and 26. Rituximab treatment induced a reduction of the mean ARR from 1.27 to 0.25 at week 24 and to 0.18 at week 72. The mean number of GAD-enhancing lesions was also reduced from 1.31 at baseline to 0.73 at week 4 after the first course and further to 0.05 at week 48 and to 0 at week 72. The mean number of new T2 lesions decreased as well, from 0.92 at week 4 to 0 at week 72, with a significant reduction also in the volume of the lesions. Rituximab was globally well tolerated: 84.6% of enrolled patients completed the week 72 visit, and all patients received the four infusions of rituximab, with the exception of one patient, in whom an IRR developed at the third infusion. The majority of enrolled patients (77%) experienced grade 1 to 2 AEs and only six reported grade 3 AEs (including fatigue, tooth fracture, muscle weakness, and headache), whereas no grade 4 events were reported. IRRs, likely due to cytokine release accompanying B cell lysis, were documented in 65.4% of the patients during the study (all mild to moderate in severity) and tended to decrease with subsequent infusions. However, no glucocorticoid premedication was administered before infusions. Infections, reported in 61.5% of patients, were also mild to moderate in severity and none led to withdrawal from the study. No opportunistic infections, including PML, were observed. None of the patients had IgG or IgA levels less than the lower limit of normal at week 72. Of the 25 patients who had normal baseline IgM values, 11 (44%) had a value below the lower limit of normal and presented a higher incidence of overall infections. Noteworthy, anti-rituximab antibodies, detected in 35% of patients at week 72, did not appear to influence either efficacy or safety measures.

In a phase II, double-blind, placebo-controlled, manufacturer-sponsored, 48-week trial (HERMES study) in 104 patients with RRMS (58), a single course of rituximab (1,000 mg on days 1 and 15) induced a drastic and sustained reduction of total GAD-enhancing lesions (relative reduction, 91%; p<0.001) and of total new GAD-enhancing lesions (p<0.001) at all investigated time points, together with a significant reduction of T2 lesions volume at week 24 (p=0.008) and 36 (p=0.004). Patients randomized to placebo had fewer GAD-enhancing lesions at baseline, but this imbalance would represent a bias against rituximab. Treatment with rituximab was also associated with a significant reduction, as compared with placebo, of the proportion of patients with relapses at week 24 (14.5% rituximab *vs.* 34.3% placebo; p=0.02) and week 48 (20.3% vs. 40.0%, p=0.04). ARR was also significantly reduced at week 24 (0.37 vs. 0.84, p=0.04), but not at week 48 (0.37 vs. 0.72, p=0.08). By week 48, CD19+ peripheral B lymphocytes, almost completely depleted from 2 weeks after treatment until 24 weeks, returned to increase. CD3+ T lymphocytes were not appreciably altered by rituximab. IgM, IgG, and IgA were normal in both groups, and IgM levels were below the lower limit of normal in more rituximab-treated patients. Sixteen (24.6%) of 65 rituximab-treated patients developed human antichimeric antibodies, although, as previously, no apparent association with the type or severity of AEs or with efficacy response at week 24, week 36, or week 48 was observed. As reported in the phase I trial, the discontinuation rate was very low, with 92.3% of the enrolled patients completing 24 weeks, and 76.0% completing 48 weeks (84.1% in the rituximab group and 60.0% in the placebo group), confirming the good tolerability of the drug in this setting. Only 6% of patients in the placebo group and 4% of patients in the rituximab group withdrew from the study because of AEs. Considering that, even in this trial, no premedication with glucocorticoids was used, 78.3% of rituximab-treated patients (versus 40.0% of patients in the placebo group) had IRRs after the first administration, mostly mild to moderate, decreased to placebo levels with successive infusions. Importantly, no differences in the rate of SAEs and infections were reported. The most common infections in rituximab group were nasopharyngitis, upper respiratory tract infections, sinusitis, and urinary tract infections, whereas no clinically significant opportunistic infections (including PML) were reported.

Globally, the efficacy results of these two trials in RRMS were encouraging and the safety evaluation was favorable: despite the high frequency of IRRs, they were mostly mild to moderate in severity, not inducing hospitalization or treatment discontinuation, and their number was reduced after subsequent infusions. Infections were also quite common in rituximab-treated patients, but, also in this case, they were mainly mild to moderate. As expected, because of the chimeric nature of rituximab, the frequency of anti-drug antibodies was higher than that reported with ocrelizumab (39, 41). However, few cases of delayed hypersensitivity reactions, associated with anti-drug antibodies forming immune complexes and observed in rituximab use for other indications (59), have been reported in MS (60). Moreover, no significant differences in treatment efficacy between the patients with and without anti-drug antibodies have been reported.

Rituximab has also been evaluated in a phase II/III randomized, double-blind, placebo-controlled, manufacturer-sponsored trial (OLYMPUS study) in patients with PPMS (61). A total of 439 PPMS patients were randomized 2:1 to receive two intravenous infusions (2 weeks apart) of 1,000 mg rituximab (n = 292) or placebo (n = 147) every 24 weeks, through 96 weeks. At week 96, treatment with rituximab compared with placebo was associated with a reduction in the proportion of patients with confirmed disease progression (CDP) — defined as an Expanded Disability Status Score (EDSS) increase of ≥1.0 (baseline EDSS 2.0 — 5.5. points) or ≥ 0.5 (baseline EDSS > 5.5. points) point from baseline values sustained for at least 12 weeks — of 8.3 percentage points (30.2% and 38.5%, respectively; p=0.14). This effect, even if not statistically significant, was quite comparable with those seen in the ocrelizumab PPMS trial ORATORIO, in which the corresponding reduction in the CDP rate compared with placebo was of 6.4 percentage points (p=0.03) (32.9% in the ocrelizumab group *vs* 39.3% in the placebo group; hazard ratio [HR]:0.76, 95% CI: 0.59–0.98) (41). Nevertheless, the prespecified subgroup analyses indicated a statistically significant effect of rituximab on CDP rate in patients younger than 51 years (HR: 0.52; p=0.010), in those with GAD-enhancing lesions at baseline (HR, 0.41; p = 0.007), and in those both younger than 51 years and with baseline GAD-enhancing lesions (HR, 0.33; p = 0.009). These results may help identify patients amenable to the treatment, with important implications for treating progressive forms of MS, for which very few therapeutic alternatives are, to date, available. Of note, ocrelizumab ORATORIO trial, in addition of having a higher sample size (488 patients in the ocrelizumab arm *vs* 292 patients in the rituximab arm) and a different statistical analysis plan, only included patients younger than 55 years (mean age, 44.7 ± 7.9 vs 50.1 ± 9.0 years in rituximab-treated patients), which may have contributed to the more favorable results obtained in this setting, supporting the approval for primary progressive MS. Moreover, with respect to rituximab-treated patients, patients in the ocrelizumab group were characterized by a shorter mean disease duration, had a higher brain volume at baseline, were slightly less disabled, and a higher percentage of them presented GAD-enhancing lesions at baseline and were treatment naïve at randomization (88.7% *vs* 64.7%) (41).

In addition, the open-label extension phase of ORATORIO trial, evaluating the effects of maintaining or switching to ocrelizumab therapy on measures of disease progression, even if demonstrating the benefit of earlier and continuous treatment with ocrelizumab over the 6.5 years of study follow-up compared with patients switching from placebo, confirmed that progression remains an important unmet need in multiple sclerosis in the long term, despite treatment with the only authorized DMT for PPMS (42).

In the OLYMPUS trial, rituximab treatment was also associated with significantly lower (p<0.001) increase in T2 lesion volume and with lower worsening in the Multiple Sclerosis Functional Composite (MSFC) timed 25-foot walk test (therefore in the ambulation) at week 96, whereas brain volume decrease was similar to placebo (p=0.62). As previously observed, rituximab induced a rapid and almost complete depletion of peripheral CD19+ B lymphocytes, which recovered at week 122 in 35% of treated patients, with no appreciable effects on CD3 T-cell counts. IgG and IgA levels were below the lower limit of normal in less than 5% of patients in either treatment arm, whereas IgM levels were below the lower limit of normal in 31.7% of rituximab-treated patients vs 5.9% of patients receiving placebo. No evidence of a relationship between lower immunoglobulin levels and an increased incidence of infections or other adverse events has been found. Twenty (7.0%) of 286 patients receiving rituximab developed human antichimeric antibodies, although, also in this case, no apparent association with the type or severity of adverse events or with efficacy responses was observed. Safety profile of rituximab reported in the trial was in line with other published data. IRRs, primarily mild to moderate in severity, were more common with rituximab (67.1% *vs* 23.1%) and decreased with successive infusions. Infections (upper respiratory infections, urinary tract infections, and nasopharyngitis) were globally reported in 65.3% of placebo and 68.2% of rituximab-treated patient, with 4.5% of rituximab *vs* <1% of placebo-treated patients reporting serious infections. AEs leading to treatment discontinuation occurred among 3% of patients who received rituximab, whereas none withdrew due to AEs was reported in the placebo group.

Despite the promising results in PPMS, as well as in RRMS, obtained from RCTs, the clinical development of rituximab was interrupted. However, in the light of the well-established long-term safety profile of rituximab from its wide use in other diseases (62) and of the promising results obtained in MS, researchers were highly motivated to pursue further trials.

A small single-center, investigator-initiated phase II trial, including 52 weeks post-treatment follow-up, evaluated the safety, efficacy, and tolerability of add-on intravenous rituximab at a dose of 375 mg/m^2^ weekly × four doses in 32 RRMS patients with breakthrough disease while receiving INFβ or glatiramer acetate (63). Enrolled patients were older, more disabled, and with a longer disease duration compared with the population of phase I and phase II placebo-controlled trial of rituximab in RRMS (57, 58). In this setting, add-on rituximab induced a significant reduction of GAD-enhancing lesions in comparison to pretreatment MRIs (p<0.0001). 74% of the three post-treatment MRI scans were free of GAD-enhancing lesions *vs* only 26% of the three pre-treatment MRIs. The median number of GAD-enhancing lesions declined from 1 per month to 0 after treatment. Although the study was not designed or powered to examine relapse rate reduction, a reduction in ARR from 1.27 pre-treatment to 0.23 after treatment has been observed. MSFC improved, mainly due to an improvement in Paced Auditory Serial Addition Test (PASAT) scores (a measure of cognitive function), whereas EDSS remained substantially stable during follow-up. As previously, also in this study, no correlation has been found between the development of human antichimeric antibodies and efficacy response. Add-on rituximab was generally well tolerated, with no SAEs and only few AEs reported. Infusion reactions, the most common AEs observed in the study, were typically mild, but resulted in two study discontinuations. Four uncomplicated urinary tract infections and one upper respiratory tract infection, with an unknown relation to rituximab, were documented.

In a more recent investigator-initiated, open-label, phase II trial (STRIX-MS trial), 75 patients with clinically stable RRMS, treated with first-line injectable IFNβ or glatiramer acetate for at least 6 months, were switched to rituximab (64). After a run-in period of 3 months, patients received two doses of 1,000 mg rituximab, followed by repeated clinical assessments, MRIs, and measurement of neurofilament light chain concentrations in the cerebrospinal fluid (NFL-CSF) for 24 months. In the first year of treatment, only one patient experienced a clinical relapse and was switched to natalizumab, whereas no patients fulfilled the prespecified MRI criteria for treatment failure (i.e., occurrence of one GAD-enhancing lesion or more than one new T2 lesions). During the second year, one patient experienced a clinical relapse and the same patient, together with three others, had a MRI worsening and was re-treated with rituximab. The mean cumulated number of GAD-enhancing lesions at months 3 and 6 and of new or enlarged T2 lesions at month 12 after treatment shift was reduced, as well as the mean CSF-NFL levels. These results support the use of rituximab in MS, given the equal or superior effect in reducing disease activity in RRMS compared to first-line treatments during the first year after switch (Class IV evidence). Regarding clinical and patient reported outcomes, there was a statistically significant improvement in the Symbol Digit Modalities Test (SDMT) (p<0.001), although the changes were small in absolute values, whereas neurologic impairment assessed by EDSS did not show any progression or improvement of statistical significance, as well as scores for patient-perceived impact of disease on daily life (Multiple Sclerosis Impact Scale, MSIS-29) and fatigue (Fatigue Scale for Motor and Cognitive functions, FSMC) (65). However, the overall treatment satisfaction, measured by a modified version of the Treatment Satisfaction Questionnaire for Medicine (TSQM-10), improved significantly, in particular for question 4 of the questionnaire (“How easy or difficult is it to use the medication in its current form?”) and question 7 (“How easy or difficult is it to live with the side effects of the medicine?”) and was sustained after 2 years. The apparent discrepancy between the improvement of patient treatment satisfaction and the lack of significant improvement in EDSS, MSIS-29, and FSMC might be explained with the overall low disability, fatigue, and therefore, global impact of the disease on daily life characterizing the patient population at the time of the switch, as well as with a more convenient treatment schedule compared to injectable first-line DMTs, with probably less interference with daily activities. Even then, the treatment was generally well tolerated. Globally, 17 non serious AEs related or possibly related to rituximab were reported. The most common side effects were, as expected, mild to moderate infusion reactions. Six SAEs were documented, three of which (two pyelonephritis and one influenza) possibly related to rituximab and three not related (stroke, cholangitis, and suicidal attempt by intoxication) (64).

A double-blind, placebo-controlled, randomized, single-center study evaluated the efficacy and safety of rituximab also as first-line treatment in an induction therapeutic approach (66). Fifty-five patients with RRMS and active disease or with a diagnosis of CIS were randomized 1:1 to receive a single cycle of rituximab (two intravenous injections of 1,000 mg 2 weeks apart) or placebo, followed by subcutaneous glatiramer acetate 20 mg/daily up to a maximum of 144 weeks. At the end of the 3 years of the study, 44% of rituximab-treated patients demonstrated NEDA *vs* 19.23% of patients in the placebo group (p=0.049). The greater probability of demonstrating NEDA in the rituximab group, observed from about 6 months from induction, was not sustained and returned to baseline within the study period. Treatment failure (defined as ≥2 new lesions, relapses, and/or sustained accumulation of disability) was observed in a smaller percentage of rituximab-treated patients (37.04% *vs* 69.23% of placebo group, p=0.019), and time to treatment failure was longer (23.32 months vs 11.29 months, p=0.027). Rituximab-treated patients demonstrated also less MRI activity as compared with placebo-treated patients, with a smaller proportion of participants having new T2 lesions (25.93% rituximab *vs* 61.54% placebo, p=0.009), and a smaller total number of new T2 lesions. No significant group differences were observed for GAD-enhancing lesions and for patient-reported outcomes regarding disability or quality of life. These results suggest that a single cycle of rituximab followed by a moderate efficacy/high safety DMT as glatiramer acetate may provide a superior efficacy than glatiramer acetate alone in RRMS, although this benefit does not seem to be long-lasting. As expected, a greater number of infusion-related reactions, all mild to moderate, was documented in the rituximab group compared with controls, whereas no differences in SAEs between the two study groups were observed.

On 2015, a phase I/II trial (RIVITaLISe) was conducted to evaluate the efficacy of combined intrathecal and intravenous rituximab therapy on SPMS compared to placebo (67). The study was prematurely terminated by investigators based on an interim analysis on CSF biomarkers that showed an incomplete and transient depletion of intrathecal B cells by rituximab. However, the early termination of the study made the acquired clinical and imaging data insufficient to perform reliable analyses of clinical effects of rituximab in SPMS patients.

Recently published results from another phase II/III, open-label, randomized clinical trial, in which 84 patients with SPMS were assigned to receive rituximab (1,000 mg every 6 months; n=37) or glatiramer acetate (40 mg subcutaneous 3 times/week; n = 40) for 12 months, documented an apparent lack of efficacy of both treatments in controlling EDSS progression (68). Indeed, the mean EDSS increased after 12 months from 3.05 ± 1.01 to 4.14 ± 0.91 in the rituximab group (p < 0.001), and from 3.22 ± 1.20 to 4.60 ± 0.67 in the glatiramer acetate group (p < 0.001). No statistically significant differences in EDSS scores were observed between the two groups, although a trend favoring rituximab emerged. In contrast, both rituximab and glatiramer acetate resulted equally efficacious in reducing ARR after 12 months (from 1.30 ± 0.52 to 0.41 ± 0.64 in the rituximab group [p<0.001], and from 1.17 ± 0.38 to 0.22 ± 0.42 in the glatiramer acetate group [p<0.001]) and the number of active lesions in brain and cervical spine. However, it has to be considered that the study had a short duration, and that patients randomized to glatiramer acetate had a longer disease (17.39 ± 7.53 years *vs* 11.41 ± 6.45, p=0.001) and were older (mean age 45.72 ± 7.64 years vs 40.92 ± 8.12, p= 0.011) compared with those assigned to rituximab group. Non-serious self-limited AEs were observed in both groups without any differences, whereas no SAEs were reported in the study.

In summary, except for the disappointing results in SPMS, even the investigator-initiated clinical trials substantially confirmed the good safety, tolerability, and efficacy profile of rituximab in RRMS, not only as second-line monotherapy but also as add-on therapy in patients not adequately controlled with first-line DMTs and as first-line monotherapy protocol (single cycle of RTX followed by other DMTs).

### Real-World Data and Retrospective Studies

Besides clinical trials, a large number of studies have used real-world data, obtained from the wide off-label use of rituximab, to assess its efficacy and safety in MS patients.

One of the largest real-world study, assessing rituximab safety and efficacy in a heterogeneous real-world MS cohort of 822 patients (557 RRMS, 198 SPMS, 67 PPMS) (69), reported a low ARR during treatment (0.044 for RRMS, 0.038 for SPMS, and 0.015 for PPMS patients) and an overall reduction of the occurrence of contrast-enhancing lesions from 26.2% at baseline to 4.6%. Most of the contrast-enhancing lesions that were detected appeared early after rituximab initiation, which eventually disappeared. The mean annual change in brain parenchymal fraction on rituximab treatment (assessed in 160 patients) was –0.19% (a percent change sensibly lower to those observed in MS patients treated with placebo in other studies) (70). During the observation time, median EDSS remained unchanged in patients with RRMS, and increased 0.5 and 1.0 for patients with SPMS and PPMS, respectively (p=0.42; p=0.10; 0.25). As previously, rituximab showed an acceptable safety profile: 7.8% of infusions led to IRRs, mostly mild, and 89 AEs grades ≥ 2 (76 infections) occurred in 72 patients. No cases of PML were detected. Treatment compliance, in line with other published data, was very high, with 94.8% of patients continuing rituximab.

Interestingly, no statistically significant differences in B-cell depletion and efficacy were reported between the two-dosing regimen used (500 and 1,000 mg doses given as single infusions every 6 months), whereas a trend for fewer AEs with the lower dose regimen was observed. These data suggest that lower doses of rituximab might be as effective in MS as higher doses with a better safety profile and a substantial cost-saving (given that the cost of rituximab is related to the dose administered).

A recently published prospective study by Disanto et al. (71), including 59 patients (37 RRMS and 22 SPMS) treated with rituximab for at least 1 year before study entry, provided evidence that the de-escalation of rituximab dose from 1,000 to 500 mg/6 months is safe and associated with clinical, radiological, and biomarker-based stability over 12 months. Indeed, no relapses were reported in the 12 months after switching to the lower dose regimen, EDSS scores maintained approximately stable, as well as serum NFL concentration, and only three new T2 lesions in brain/spinal cord (all of which without contrast enhancement and clinically asymptomatic) were detected. Such a result is striking considering that most of the included patients had a severe form of MS and started rituximab mainly because of the suboptimal response on previous DMTs. Overall, three SAEs, only one (a late-onset transient neutropenia) probably related to rituximab, occurred in the 12 months after dose de-escalation. The most common AEs were infections, whereas no IRRs were reported after dose switching. A greater risk of infections was detected in those patients with a mean IgG concentration below the reference range (OR=6.27, 95% CI=1.71–22.9, p=0.005). Importantly, an inverse association between the total dose of rituximab received under the 1,000 mg/6 months regimen (rituximab load) and the IgG concentrations measured after the de-escalation emerged in the study, with a higher rituximab load associated with a lower IgG, and, therefore, with a greater risk of infections.

Another huge multicenter, retrospective Italian-Swiss study, analyzing data from over 350 RR and progressive MS patients treated with rituximab, showed a significant reduction of ARR in the 2 years after the treatment start from 0.86 (95% CI: 0.73–0.99) to 0.09 (95% CI: 0.07–0.13) in RRMS and from 0.34 (95% CI: 0.25–0.45) to 0.06 (95% CI: 0.04–0.10) in SPMS patients (p<0.0001), and a slight not significant decrease in PPMS patients (from 0.12 to 0.07, p = 0.45) — probably related to the lower number of events (72). The proportion of patients with an EDSS progression was 14.6 ± 0.07% in the RRMS group, 24.7 ± 0.11% in the SPMS group, and 41.5 ± 0.17% in the PPMS group, after 3 years of treatment. In the multivariable analysis, the risk of EDSS progression was higher for PPMS (p=0.0005) and SPMS (p=0.013) as compared with RRMS patients. AEs observed during rituximab treatment were within the expected range, including mostly IRRs and infections, both rarely reported to be serious. No major safety concerns (especially those related to neoplasms or PML) arose. Overall, the study adds to the published literature, confirming that rituximab is effective and relatively safe in the treatment of MS.

An interesting propensity score matching analysis performed on data retrospectively collected from three MS centers located in Switzerland and the Netherlands, showed, in contrast to what reported in the phase II/III trial in SPMS (68), a significantly lower EDSS score during a mean follow-up of 3.5 years (mean difference, −0.52; p<0.001) and a significantly delayed time to confirmed disability progression (p=0.03) for patients treated with rituximab compared with matched patients never treated with rituximab, suggesting a potential therapeutic benefit of rituximab also in SPMS (73). No major safety concerns were reported during the treatment period, although complications, mainly related to infections, were documented in five cases (9%).

A single-center retrospective observational study in Finland (74), included a total of 72 rituximab-treated patients with RRMS (n=31), PPMS (n=16), and SPMS (n=25) for whom other MS medications failed to achieve an adequate effect, or for whom no other medication was available. EDSS remained substantially stable in all MS group. In particular, among patients with progressive forms, 45% had stable EDSS during the study, whereas 18% of PPMS and 20% of SPMS patients even had an improvement. Moreover, rituximab treatment significantly reduced ARR in both RRMS and SPMS and the mean number of GAD-enhancing lesions in RRMS patients. Treatment discontinuation was observed in 12 patients because of the patient’s disappointment with the drug efficacy (n=10) or a drug-related adverse event (n=2). The study confirmed the good tolerability of rituximab, also in this setting, with no serious IRRs or infections.

A large cross-sectional study by Dunn and collaborators (75), including patients receiving off-label rituximab for MS (both RR and progressive forms), reported the development of anti-rituximab antibodies in 34% of patients (a percentage higher to that observed in clinical trials). The presence of anti-drug antibodies, which decreased after repeated rituximab infusions, was associated with incomplete or unmaintained B-cell depletion, but not with infusion reactions, adverse events, or lack of clinical effect, with a strong suppression of disease activity observed in both antibody-positive and antibody-negative patients.

A retrospective observational study (76), based on data collected within a registry, provided further evidence of the efficacy of rituximab in MS treatment, both in RRMS and PMS in terms of number of new relapses, EDSS worsening, new T2 and GAD+ lesions, and proportion of patients without evidence of disease activity during treatment.

A small retrospective study confirmed the good tolerability and acceptable safety profile of rituximab also after long-term treatment (average duration, 33.2 months) (77). AEs reported during the observation period were mostly mild, with the exception of three severe urinary tract infections requiring hospitalization, and no cases of PML.

Effectiveness and safety of rituximab were further confirmed in a recent Italian single-center retrospective observational analysis of 17 patients with demyelinating CNS diseases (including MS, neuromyelitis optica, and neuromyelitis optica spectrum disorders [NMOSD]) who underwent rituximab treatment (78). About 25% of patients were naïve to DMTs. The mean follow-up was 22.6 ± 22.9 months (range, 12–80 months). After rituximab treatment, 11 (65%) of 17 patients got NEDA status, and no patients had disability progression and new T2 or T1-GAD+ brain and/or spinal lesions. Six AEs were recorded in five patients. One patient with RRMS stopped rituximab and switched to azathioprine due to severe lymphopenia, whereas another patient with PPMS switched to ocrelizumab after its license for PPMS treatment.

In another retrospective study on an Italian real-life cohort of RR and progressive MS patients, the most of which not treatment naïve and switched to off-label rituximab due to persistent disease, AEs or reduced compliance, rituximab (1,000 mg, 6 monthly) significantly reduced the ARR from 0.75 to 0.36 at 12 months (p<0.001), with no differences between RR and progressive patients (79). The proportion of patients showing MRI activity was reduced from 88% to 8.3% at follow-up (p<0.001), again with no differences between RR and progressive patients. Of the 55 patients who had an EDSS evaluation, 13 (23.2%; 10 PMS, and 3 RRMS) showed a progression at 6 months compared with baseline, whereas only one progressive patient showed a progression at 12 months. The NEDA status at 12 months was observed in about 60% of patients. The reported safety profile in this patient group was substantially consistent to that reported in other studies, with a high frequency of mild-to-moderate IRRs and infections. Interestingly, infectious AEs were less common than non-infectious and 10% of the reported AEs were leukopenia. Globally, 12 patients suspended rituximab during the study due to AEs (n=4), scarce tolerability (n=3), persistent clinical (n=2) or radiological disease activity (n=2), or pregnancy (n=1).

A retrospective cohort university hospital-based study (80), analyzing data from 59 RRMS and 30 PMS patients switched to rituximab mainly due to persistent disease activity on other DMTs, showed a reduction of ARR by approximately 89% (relapse-free in 79% in the RRMS and 90% in the PMS group) and no EDSS score progression in both RRMS and PMS patients. Interestingly, there was a trend of improvement in terms of EDSS in RRMS, whereas in the PMS group, it was substantially unchanged. 92.6% in the RRMS and from 82% in the PMS group were free from any new lesions, and 74% achieved NEDA at 1 year of treatment. The most common AEs (n=64; 71.9%) were mild IRRs, whereas the overall rate of infection was relatively low (15.7%). Two rituximab-treated patients (2.2%) experienced SAEs requiring surgical interventions (pyoderma gangrenosum vaginalis with perianal abscess and fistula; increase in the size of a meningioma). No cases of PML were reported.

In another Spanish retrospective university hospital-based study, including both RRMS and PMS patients, rituximab (administered off-label mainly as second- or third-line treatment) significantly reduced ARR by 88.4% (p<0.001) and the number of GAD-enhancing lesions from 2.56 to 0.06 (p<0.001) (81). Ninety percent of patients remained free of relapses during the follow-up and the relapses observed in the remaining patients occurred almost all in the first 6 months of treatment. A decrease of 0.3 EDSS points in the first year (p=0.01) and no variation in the second year of therapy were detected. Considering only PMS patients, most of them remained stable after rituximab treatment, without significant changes in the EDSS score. NEDA status was reached in 70% of the total sample (74.2% of RRMS patients, and 67% of the PMS patients). Therefore, in this study, rituximab demonstrated to be a feasible therapeutic option for PMS patients as well. The main AEs were IRRs, mostly mild and less frequent than those reported in clinical trials (18.8% vs 60–70%). Regarding non–infusion-related AEs, the most common were non-severe infections, while no opportunistic infections like PML were reported. One case of agranulocytosis 3 months after rituximab infusion and three cases of venous thrombotic events (one deep venous thrombosis in one leg, one deep venous thrombosis with secondary mild pulmonary embolism in a patient taking concomitant oral contraceptives, and a serious massive pulmonary embolism secondary to a deep venous thrombosis in a patient with an EDSS score of 8.5 and lack of mobility) were reported. Rituximab was interrupted in 22 (24.4%) patients, mainly as a consequence of suboptimal responses or disability worsening (especially in PMS patients).

At a general hospital level, Hellgren et al., retrospectively analyzing data from 83 patients with RRMS, PPMS, and SPMS, reported a highly significant reduction of ARR induced by rituximab (500 or 1,000 mg every 6–12 months) from mean 0.38 ± 0.5 before treatment initiation to mean 0.05 ± 0.19 at follow-up (p<0.00001), with a global reduction by 87% (82). The percent of patients with new inflammatory lesions decreased from 58% at baseline to 26% during the long-term follow-up, from 36 to 18 (p<0.0001) in the RRMS cohort, and from 8 to 2 (p=0.07) in PMS. Considering only contrast-enhancing lesions, the percent of subjects with one or more lesions dropped from 47% at baseline to 6% at 1 year after rituximab initiation. Globally, contrast-enhancing lesions decreased from 0.94 to 0.24 (p<0.00001). In the RRMS cohort, contrast-enhancing lesions/MRI ratio was reduced from 1.05 to 0.31 (p=0.00003), whereas no lesions were seen in the PMS patients after rituximab initiation. The most interesting finding of this study was that most scans showing contrast enhancement were done within 6 months after starting rituximab, whereas a total absence of new lesions was reported in almost all patients during the remaining follow-up period (mean duration ~2 years). Reported AEs were mainly mild. Most frequent non-IR AEs were infections (observed in 22% of treated patients), of which four were classified as moderate, requiring hospitalization, and one as severe (a case of pneumonia with concomitant late-onset neutropenia, the first reported in Swedish MS population related to rituximab).

An interesting retro-prospective study performed in a developing country (India), where rituximab, also thanks to the availability of biosimilars, represents an affordable therapeutic option for MS with respect to other high-cost approved standard treatments, demonstrated the good safety and efficacy profile of three different dosing regimens (a low-, a medium-, and a high-intensity regimen, chosen depending on the severity of MS) of the DMT in RRMS (n=58) and PMS (n=15 SPMS and n=7 PPMS) patients (83). In the RRMS population, the mean ARR decreased from 0.44 ± 0.498 to 0.051 ± 0.223 (p<0.05) at 1 year of follow-up, with no relapses in 97% of treated patients. EDSS improved by 0.5 to 2.0 points in 85% of patients (all RRMS patients, four SPMS and six PPMS), remained stable in 12.5% (9 SPMS and 1 PPMS), and worsened in 2.5% (2 SPMS patients). In all treated patients with GAD-enhancing lesions at baseline, follow-up scans at 1 year did not show any lesions either old or new. Interestingly, in the study, the incidence of IRRs was minimal, probably as a consequence of the very low infusion rate adopted (64 ml/h). As in the other real-world studies, no opportunistic infections, like tuberculosis or PML, were reported.

A retrospective study, involving 29 patients with immune-mediated neurological disorders (MS, neuromyelitis optica, and myasthenia gravis) treated with rituximab for up to 7 years (mean treatment duration of 51.3 ± 12.2 months) confirmed the long-term safety and efficacy of rituximab in this setting (84). A total of 32 AE and 4 SAEs (all infections in both cases) were reported, whereas no cases of PML or tumors were detected over the observation period. Rituximab cycles resulted globally well tolerated, with minimal and manageable IRRs, and an overall benefit in terms of relapse rate reduction and improvement in EDSS was observed. Another recent large retrospective study (85), including 1,000 patients with MS, NMOSDs, and other immunological disorders with a mean follow-up of 31.1 months, reported a low incidence of serious AEs, especially infections, associated with rituximab. The overall rate of infections, resulting in hospitalization, intravenous antibiotics, and extended dosing antibiotics, was nearly identical to that reported in a long-term study of rituximab-treated RA patients (86). No cases of PML were observed. IRRs reported in the study were rarely serious, with no infusion deemed life-threatening or resulting in hospitalization, and the rate of malignancy was similar to those of the general population (87). Interestingly, a dramatic increase in infection risk was reported for patients with increasing levels of ambulatory disability, highlighting the importance of using rituximab in younger, less disabled patients early in the disease.

The good safety and efficacy profile of rituximab in both relapsed and progressive forms of MS have been confirmed by different meta-analysis. A meta-analysis by Hu et al. (88), including 15 studies and a total of 946 patients with RRMS, showed a significant decrease of ARR, of EDSS score, and a low percentage of patients experiencing a relapse after starting rituximab therapy. Although mild-to-moderate AEs (mainly infusion-related events and infections) occurred in 29.6% of the patients, no SAEs were reported.

A more recent meta-analysis, including 20 studies for a total of 2020 RRMS patients, reported even more favorable results, with an overall absolute reduction in ARR of 1.00 (95% confidence interval [CI], 0.83–1.17), an overall relapse-free rate at weeks 24, 48, 72, and 96 of 90.4%, 88.5%, 86.4%, and 86.2%, respectively, and an estimated reduction in EDSS score of 0.62 (95% CI, 0.20–1.04) (89). overall AEs (58%; 95% CI, 12%–104%), injection-related events (31%, 95% CI, 18%–45%), and infections (33%; 95% CI, 20%–46%) were common in patients treated with rituximab, whereas SAEs were rarely reported, confirming an acceptable safety profile of rituximab.

Another meta-analysis, including seven studies for a total of 399 patients with any type of MS treated with rituximab, showed a reduction of mean EDSS score (0.29; 95% CI, 0.16–0.42) and of mean ARR (1.24; 95% CI, 1.04–1.44) after treatment, and a proportion of AEs (mostly infusion-related reactions and infections) of 23% (95% CI, 20%–26%) (90).

### Indirect Comparisons

Currently, no head-to-head RCTs comparing rituximab with other DMTs have been completed. **Table 2** provides a list of ongoing clinical trials comparing DMTs, including rituximab.

**Table 2:** Ongoing clinical studies comparing disease modifying therapies, including rituximab (www.clinicaltrial.gov; update January 2021).

ID	Title	Trial design	Age (years)	Number of estimated patients (disease)	Arms and interventions	Primary Outcome measures	Follow-up duration	Start date/Estimated Study Completion Date
NCT03500328	A Pragmatic Trial to Evaluate the Intermediate-term Effects of Early, Aggressive Versus Escalation Therapy in People With Multiple Sclerosis	Phase NA, randomized, parallel assignment	18-60	900 (RRMS)	*Early aggressive therapy choices*: -natalizumab; -alemtuzumab; -ocrelizumab; -rituximab; -cladribine; -ofatumumab *Traditional therapy choices*: -glatiramer acetate; -intramuscular interferon; -subcutaneous interferon; -pegylated interferon; -dimethyl fumarate; -fingolimod; -siponimod; -ozanimod.	-Time to sustained disability progression; -change in overall burden of MS	63 months	May 2, 2018/August 1, 2023
NCT04047628	A Multicenter Randomized Controlled Trial of Best Available Therapy Versus Autologous Hematopoietic Stem Cell Transplant for Treatment-Resistant Relapsing Multiple Sclerosis	Phase III, randomized, parallel assignment	18-55	156 (MS with EDSS ≥ 2.0 and ≤ 5.5, excluding PPMS)	-Myeloablative and Immunoablative therapy followed by Autologous Hematopoietic Stem Cell Transplantation - Best Available Therapy (BAT) Natalizumab, alemtuzumab, ocrelizumab, or rituximab
NCT03535298	Determining the Effectiveness of earLy Intensive Versus Escalation Approaches for the Treatment of Relapsing-Remitting Multiple Sclerosis	Phase IV, randomized, parallel assignment	18-60	800 (RRMS)	-*Early highly effective* arm: ocrelizumab, natalizumab, alemtuzumab, rituximab)- *Escalation arm*: any other approved MS therapy (beta interferon, glatiramer acetate, teriflunomide, fingolimod, dimethyl fumarate) - *No intervention*	Brain volume loss	36 months	January 3, 2019/September 2023
NCT02746744	RItuximab Versus FUmarate in Newly Diagnosed Multiple Sclerosis. A Randomized Phase 3 Study Comparing Rituximab With Dimethyl Fumarate in Early Relapsing-Remitting Multiple Sclerosis and Clinically Isolated Syndrome.	Phase III, randomized, parallel assignment	18-50	200 (RRMS)	-Rituximab -Dimethyl Fumarate -Sham comparator	Freedom of relapse	2 years	May 2016/August 2021
NCT04121403	Norwegian Study of Oral Cladribine and Rituximab in Multiple Sclerosis (NOR-MS) A Prospective Randomized Open-label Blinded Endpoint (PROBE) Multicenter Non-inferiority Study	Phase III, randomized, parallel assignment, open-label blinded endpoint (PROBE) multicenter non-inferiority study	18-65	264 (active RMS)	-Rituximab -Cladribine	Number of new or enlarging cerebral MRI T2 lesions	96 weeks	October 16, 2019/December 2023
NCT03193866	COMparison Between All immunoTherapies for Multiple Sclerosis. An Observational Long-term Prospective Cohort Study of Safety, Efficacy, and Patient’s Satisfaction of MS Disease Modulatory Treatments in Relapsing-remitting Multiple Sclerosis	Prospective non-intervention observational prospective cohort	≥ 18	3526 (CIS or RRMS)	-Rituximab -all other frequently used immunomodulating (natalizumab, fingolimod, alemtuzumab, interferon-beta, glatiramer acetate, dimethyl fumarate)	Confirmed disease progression in patients with EDSS ≤2.5 at baseline	3 years	February 1, 2017/December 31, 2022
NCT04688788	Danish Non-inferiority Study of Ocrelizumab and Rituximab in MS (DanNORMS): A Randomized Study Comparing the Efficacy of Ocrelizumab and Rituximab in Active Multiple Sclerosis	Phase III, randomized, open-label, non-inferiority clinical trial with blinded primary endpoint.	18-65	594 (RRMS, PMS)	-Biosimilar rituximab -ocrelizumab	Percentage of patients without new or enlarging T2 white matter lesions on brain MRI scans	24 months	January 15, 2021/January 15, 2028
NCT04578639	Ocrelizumab Versus Rituximab Off-Label at the Onset of Relapsing	Phase III, randomized double blinded non-inferiority study	18-60	211 (active RRMS)	-rituximab -ocrelizumab	Proportion without new MRI activity	24 months	November 2, 2020/February 14, 2025

CIS, clinically isolated syndrome; EDSS, expanded disability status score; MRI, magnetic resonance imaging; MS, multiple sclerosis; NA =; PPMS, primary progressive multiple sclerosis; PMS, progressive multiple sclerosis; RMS, relapsing multiple sclerosis; RRMS, relapsing remitting multiple sclerosis; NA, Not Available.

However, real-world studies have allowed to carry out indirect comparisons (76, 91–95). A propensity score–matched Swedish registry study (95), assessing efficacy of rituximab (n=461) in comparison with interferons/glatiramer acetate (n=922), demonstrated a superiority of rituximab over injectable DMTs in the reduction of ARR and EDSS from baseline to 12 and 24 months. Rituximab was also associated with an 85% reduction in the rate of discontinuation relative to IFN-β/glatiramer acetate (HR, 0.15; 95% CI, 0.11–0.20).

The retrospective cohort study by Granqvist et al. (93), including 494 Swedish patients with newly diagnosed RRMS, found a significantly lower discontinuation rate with rituximab (500 mg or 1,000 mg intravenous every 6 months) compared with all other DMTs included in the analysis (interferons, glatiramer acetate, dimethyl fumarate, fingolimod, and natalizumab). The most common cause of treatment discontinuation was pregnancy for rituximab, disease breakthrough and AEs for injectable DMTs, dimethyl fumarate, and fingolimod, and positive JCV serology for natalizumab. Regarding clinical efficacy and safety, a significantly lower rate of relapses and/or disease activity was found with rituximab together with a lower incidence of AEs compared with injectable DMTs and dimethyl fumarate. Compared with fingolimod and natalizumab, ARR and GAD+ lesions were numerically lower but did not reach statistical significance.

Boremalm et al. (94), in a small cohort of 241 RRMS patients switched from interferon/glatiramer acetate due to breakthrough disease, found no significant difference in ARR between natalizumab and rituximab (HR, 1.0; 95% CI, 0.2–5.6), both before and after adjustment for confounders. Both natalizumab and rituximab demonstrated superiority compared with fingolimod, in terms of clinical efficacy. As previously, the discontinuation rate was significantly lower with rituximab compared with both natalizumab and fingolimod. The comparable efficacy of rituximab and natalizumab was further supported by the aforementioned retrospective study by Scotti et al. (76), reporting a similar disease activity reduction in RRMS patients both in multivariate Cox models and after propensity score-based matching.

Globally, these studies showed a greater drug survival and tolerability of rituximab and an efficacy, in terms of control of relapses and MRI activity, comparable to natalizumab and superior to dimethyl fumarate and fingolimod, although considerable variability was observed in the magnitudes of the reported differences.

A recent retrospective US study, which performed a head-to-head comparison between 182 rituximab-treated patients and 1,064 patients who received dimethyl fumarate, fingolimod, or natalizumab over 2 years after treatment initiation (91), demonstrated decreased odds of discontinuation and improved efficacy for rituximab compared with fingolimod and dimethyl fumarate, whereas no significant differences were observed between rituximab and natalizumab. However, when investigation of disease activity was restricted between months 6 and 24, an improved effectiveness of rituximab over natalizumab has been reported. Notably, although rate of discontinuation was similar, rituximab discontinuations were driven by insurance issues related to the off-label use, whereas natalizumab discontinuation was mainly related to safety issues.

A new retrospective study, comprising RRMS and SPMS patients treated with rituximab (n=311) and RRMS patients treated with ocrelizumab (n=161), compared tolerability, safety, and immunosuppressive effects of the two anti-CD20 drugs over the first year of treatment (45). The researchers found that ocrelizumab, but not rituximab, was associated with a decrease in IgG of 0.16 g/L (95% CI, 0.01–0.31) with each infusion (a reduction that may increase susceptibility to infections), whereas IgM decreased to a similar extent with both drugs and IgA levels were not affected. CD19+ B depletion was greater with ocrelizumab. Infections and SAEs were more common in the ocrelizumab group, whereas incidence of IRRs was identical to that of rituximab. No statistically significant differences were observed in the proportion of patients discontinuing treatment within the first year (10% with rituximab and 15% with ocrelizumab, p=0.11). However, although the discontinuation due to lack of effect was low and not significantly different in the two groups, discontinuation due to AEs was more common with ocrelizumab than with rituximab. These findings corroborate the idea of the non-inferiority, in terms of tolerability and safety, of rituximab to ocrelizumab, and substantially confirm that the development of anti-drug antibodies, higher with the more immunogenic rituximab and potentially associated with reduced efficacy and increased risks of IRRs, is of marginal clinical importance.

Another recent study analyzed AEs reported for rituximab and ocrelizumab in the real-world practice setting using the Food and Drug Administration’s Adverse Event Reporting System (FAERS) database (96). The database contained 623 reports with rituximab and 7948 reports with ocrelizumab. Patients treated with rituximab were on average older than patients treated with ocrelizumab and progressive forms of MS were more frequently found in the reports associated with ocrelizumab (21.2% *vs* 5.6%). Rituximab was associated with a higher proportion of reported SAEs as compared to ocrelizumab (64.8% *vs* 56.3%, p < 0.001). AEs resulting in death were found in 5.7% of rituximab reports versus 2.1% of ocrelizumab reports (p < 0.001). The study revealed significant differences in reported AE profiles in the real-world setting between the two anti-CD20 drugs, with frequency of reported infections (especially oral herpes, urinary tract infections, and nasopharyngitis) nearly two times higher with ocrelizumab (21.93% *vs* 11.05% of rituximab), whereas no significant differences were reported for IRRs. However, the risk of bias in spontaneous reporting system, above all under-reporting and the tendency for SAEs to be reported more frequently, should be considered.

Preliminary results from an ongoing phase III trial (ClinicalTrials.gov Identifier: NCT02980042), evaluating tolerability and safety of switching from rituximab to ocrelizumab in adult patients with relapsing forms of MS (97), reported a similar incidence of IRRs between patients continuing rituximab and those switched to ocrelizumab and suggested a correlation between levels of CD19/CD20 B cells and risk of IRR (with a decrease by 74% of the risk when CD19 and/or CD20 were ≤1%).

Perez et al. confirmed the similarity of rituximab originator and biosimilar in 145 MS patients (RR and progressive) (98). Patients in the two groups did not differ in CD19+ lymphocyte counts at each follow-up examination and showed a comparable reduction in relapse rate at 12 months (from 0.50 to 0.02 for originator and from 0.40 to 0.025 for biosimilar), whereas EDSS remained stable in both groups at 6 and 12 months. The proportion of patients with MRI activity on the first scan after starting of rituximab was similar between originator and biosimilar (1% and 0% of patients with GAD-enhanced lesions, respectively, p=0.41; 10% and 12% of patients with new T2 lesions, respectively, p=0.76). On the second MRI after rituximab initiation, only one patient in the entire population (treated with originator) showed a new T2 lesion, whereas no new GAD-enhanced lesions were detected. AEs were also similar, with mild-to-moderate IRRs being the most frequent AEs. No severe or opportunistic infections were reported, and no patients discontinued rituximab after 1 year.

Different studies have also assessed rituximab after switching from another DMT in real-world populations. In a retrospective study by Alcalá et al., rituximab has proven to be an effective and safe therapeutic alternative in a small cohort of RRMS patients after fingolimod withdrawal due to suboptimal response or side effects, with an efficacy profile comparable to that of alemtuzumab (99). The ARR was significantly reduced by rituximab with no statistical differences from what was observed with alemtuzumab. Similarly, the median EDSS was significantly reduced with rituximab, without statistical differences compared with alemtuzumab. No difference was detected as regard to patients reaching NEDA. Rituximab, as well as alemtuzumab, was also safe in the study cohort, with reported AEs consistent with that already described in the literature.

Another small retrospective study, including 12 patients with RRMS — all of which had failed first-line therapy (IFN and glatiramer) and seven of which had also failed second-line therapy (natalizumab/fingolimod) — confirmed rituximab as a safe and effective second- or third-line DMTs, even in patients (n=2) who developed a concomitant autoimmune disease (idiopathic thrombocytopenic purpura) during the course of MS (100). During the follow-up period (mean duration 40 months), no patients switched to rituximab experienced SAEs or discontinued treatment. No patients had a clinical relapse, MRI activity was not detected and the EDSS scores improved in 11 of 12 patients and remained stable in one patient. Furthermore, an improvement of EQ VAS score, and thus an improvement in patient-perceived health status, has been reported almost in all treated patients.

A French nationwide retrospective multicenter study demonstrated the efficacy of off-label rituximab as rescue therapy in 50 patients with active RRMS despite immunosuppressive DMT (fingolimod, natalizumab, or mitoxantrone) (101). The median total number of previous treatments was 3 (range, 2–6), and the median number of immunosuppressive DMT was 2 (range, 1–3). The ARR was significantly reduced by rescue therapy with rituximab from 0.8 during last immunosuppressive DMT to 0.18 (p<0.0001), and almost all rituximab-treated patients showed a stable or decreased EDSS score at the last clinical evaluation (p<0.0001). The percentage of patients showing contrast-enhancing lesions was also significantly reduced from 72% to 8% after rituximab initiation (p<0.0001). Interestingly, almost 95.5% of the MRI performed after rituximab initiation did not show any inflammatory activity. 70% of included patients reached NEDA status at the last clinical evaluation (median, 1.1 years; range, 0.5–6.4 years). The safety profile of rituximab was in line with other observations, with IRRs and infections as the most common AEs, and no cases of PML reported.

In a cohort of 10 patients with RRMS that stopped natalizumab treatment due to high risk of PML, the switch to rituximab resulted efficacious in preventing disease reactivation or rebound and in maintaining radiological stability (102). Rituximab resulted to be a valid post-natalizumab treatment option, with no new relapses recorded, also in small cohort of 16 MS patients switched from natalizumab because of positive JCV serology (76). In another cohort of RRMS patients from two Italian centers who interrupted natalizumab after at least six infusions and with a follow-up of at least 12 months, no evidence of disease reactivation was observed in those switched to off-label rituximab (103). In contrast, clinical and/or radiological reactivation was observed in patients switched to first-line therapies (IFNβ, glatiramer acetate, teriflunomide, azathioprine), fingolimod, and immunosuppressive agents (cyclophosphamide or mitoxantrone).

In a cohort of 256 stable RRMS patients who switched from natalizumab solely due to JCV antibody positivity (92), rituximab showed a better risk-benefit profile compared with fingolimod (the most studied post-natalizumab therapy). In particular, the rituximab-switched group experienced less relapses, fewer contrast-enhancing lesions, and less drug discontinuations compared with fingolimod-switched patients. Regarding discontinuations, most of them in the fingolimod group were due to disease breakthrough, highlighting the higher effectiveness of rituximab. Furthermore, the hazard ratio (favoring rituximab) for AEs (5.3% in rituximab group *vs* 21.1% in fingolimod group) was 0.25 (95% CI: 5 0.10–0.59), indicating a better tolerability of rituximab despite a higher rate of first-dosing AEs compared with fingolimod (26% *vs* 7%).

More limited data are, to date, available on the switch from natalizumab to ocrelizumab. Seven cases of PML have been reported in patients treated with ocrelizumab after natalizumab, therefore, this switch may not be safe (104). However, in a retrospective analysis on 28 patients switched from natalizumab after a median washout period of 44 days (35–83 days), ocrelizumab has been proven to be safe and effective, with absence of new relapses and no cases of PML, although, as for rituximab, the emergence of PML that could elude MRI detection remains a potential concern (105). In another retrospective analysis on 42 RRMS patients who switched from natalizumab to ocrelizumab due to high risk of PML, despite a disease reactivation in 12% patients in the first 3 months, no further relapses were observed with ocrelizumab, EDSS remained stable in 90% of cases and no carryover PML nor significant AEs occurred (106).

Finally, some case reports confirm rituximab to be a safe and effective treatment in controlling MS reactivation after natalizumab interruption. Recently, the case of a young woman, who interrupted natalizumab treatment due to PML diagnosis, has been described (107). After the interruption of natalizumab, the patient experienced an important clinical worsening (EDSS worsened from 4 to 8) and multiple new lesions in the brain and spinal cord. After fingolimod failed to control this MS reactivation, rituximab was started, inducing a dramatic improvement in patient’s clinical conditions (EDSS 5.5, no relapses or MRI activity) and no reactivation of PML occurred.

### Special Population

Some real-world studies have also suggested that rituximab may represent an optimal therapeutic choice, even superior to other DMTs, for women with MS planning a pregnancy.

Indeed, the management of MS in pregnant women remains challenging due to the lack of approved DMTs for use in this population, and the risk of rebound after discontinuation of certain DMTs. The risk of rebound after discontinuation has not been reported with ocrelizumab (108). However, little is known about the safety profile of ocrelizumab in pregnancy, and current guidelines recommend contraception for women of childbearing age while receiving ocrelizumab and for 6 months after the last infusion of ocrelizumab, given the unknown fetal risk (109).

A large observational cohort study, including 586 women with MS onset, before childbirth identified through the Swedish MS Registry, showed a relapse rate 1 year post-partum significantly higher in women who suspended natalizumab within 6 months before conception and in women untreated within 1 year before conception compared with women who suspended rituximab in the 6 months before conception (adjusted rate ratio [aRR], 7.65; 95% CI, 2.47–23.6 and 4.69; 95% CI, 1.67–13.2, respectively) (110). Moreover, in the suspended rituximab women, only one maternal relapse occurred during pregnancy and only one of four patients who relapsed in the first quarter after delivery experienced new GAD+ lesions. These results suggest a prolonged protective effect on MS disease activity of rituximab, which can encompass pregnancy and postpartum period, without the high risk of disease reactivation or rebound described with natalizumab withdrawal before pregnancy (111). In line with these data, a German cohort study (112), analyzing 88 pregnancies from 81 women with neuroimmune diseases (including MS and NMOSDs) treated with anti-CD20 mAbs in the year before conception, showed a good control of disease activity during pregnancy and postpartum, with no major safety concerns (with the exception of two congenital abnormalities reported in women exposed to ocrelizumab during pregnancy) and with pregnancy outcomes within the range expected for the general population. An interesting case series about 11 pregnancies in 10 women (7 with MS and 3 with NMOSDs) treated with rituximab within 6 months of conception, seems to confirm these safety and efficacy findings: indeed, all completed pregnancies resulted in term live births of healthy newborns, no maternal relapses occurred before/during pregnancy and only one was observed in the post-partum (113).

An interesting case report documented the high efficacy and safety of rituximab in controlling a severe rebound in a woman with MS who interrupted fingolimod during the first month of pregnancy (114). Eight weeks after withdrawal of fingolimod, the patient developed severe symptoms resulting from multiple new and enlarging lesions and a significant worsening of EDSS (from 3.0 to 7.0). Considering the severity of her conditions and to prevent further relapses, rituximab was started at week 22 of gestation and continued during the rest of the pregnancy and beyond. No new relapses occurred, and by the end of the pregnancy, she partially recovered from disability. No adverse fetal or infant effects were reported as the patient delivered, at 38 weeks of gestation, a healthy boy (APGAR score 9 at 1 min, and 10 at 5 min) with a normal 3-month development.

## The Regulatory Perspective: Off-Label Use

To date, rituximab is authorized for various therapeutic indications, including the following onco-hematologic and auto-immune diseases: non-Hodgkin’s lymphoma (NHL), chronic lymphocytic leukemia (CLL), rheumatoid arthritis (RA), granulomatosis with polyangiitis, and microscopic polyangiitis, Pemphigus vulgaris. This anti-CD20+ antibody, with the same mechanism of action as ocrelizumab, should be also considered as a therapeutic option for MS patients, although it does not hold regulatory approval for this indication, given its good and well-known efficacy and safety profile, emerging from clinical trials and the wide real-world use as monotherapy for RR and progressive forms. Therefore, the prescription in patients with multiple sclerosis is a typical off-label use, “not in accordance with the authorized product information” (115). This off-label use is common, not only as an escalation therapy but also as a first-line treatment. For example, rituximab is the most commonly used DMT in Sweden for all MS subtypes, although with considerable regional differences (116). Moreover, differently from ocrelizumab added to the repertoire of MS therapies around 2017 to 2018 and with limited post-marketing use, long-term safety of rituximab is well documented not only in MS but also in other conditions, such as rheumatoid arthritis, where prolonged exposure for 11 years was well tolerated and not associated with increased safety risks, including serious opportunistic infections and PML (86). Finally, it has a more favorable price with respect to ocrelizumab, even considering the availability of different biosimilar versions. For example, if we consider the Italian prices, the estimated expenditure with the available RTX products (calculated for a maximum dosage of 1,000 mg*2 and 2 cycle/year -1 cycle every 6 months) are reduced by more than half compared to ocrelizumab (**Table 3**).

**Table 3:** Estimated expenditure for anti-CD20 in MS in Italy.

Pharmaceutical Product	Dosage form	Packaging	Price/box*	Cost/Cycle (max)^§^	Cost/Year (max)°
**Rituximab**					
**Mabthera^®^**	500 mg concentrate for solution for infusion	1 vial 50 mL	€ 1.252,41	€ 5.009,64	€ 10.019,28
**Rixathon^®^**	500 mg concentrate for solution for infusion	1 vial 50 mL	€ 1.001,93	€ 4.007,72	€ 8.015,44
**Truxima^®^**	500 mg concentrate for solution for infusion	1 vial 50 mL	€ 1.110,17	€ 4.440,68	€ 8.881,36
**Ocrelizumab**					
**Ocrevus^®^**	300 mg concentrate for solution for infusion	1 vial 10 mL	€ 5.640,63	€ 11.281,26	€ 22.562,52

*ex factory price (source www.codifa.it).

**^§^**RTX max 1.000 mg*2.

°RTX max 2 cycle (1 cycle every 6 months).

Currently, off-label use is not regulated in Europe, but some member states adopted specific national measures (115, 117). For example, the France *Recommandations Temporaires d’Utilisation* (RTU) (118) and the Italian Law 648/1996 (119, 120) ensure a nationwide access to off-label drugs according to criteria for appropriate use and monitoring defined in the light of clinical evidence (at least phase II trials for 648/96). In both cases, public bodies (patient associations, scientific societies, clinical centers) may submit to the national competent authority the requirement for the approval of an off-label use of a medicinal product.

These laws permit to recognize the therapeutic use of effective and safe medicines beyond the interest of pharmaceutical companies for new extension of indications.

Rituximab received a RTU in 2018 for the treatment of patients with severe Immune Thrombocytopenic Purpura (ITP), refractory to other treatments.

Moreover, Italy allows to reimburse the drug for the following off-label use in accordance with Law 648/1996:

– HCV-related mixed cryoglobulinemia refractory to antiviral therapy, HCV-related mixed cryoglobulinemia with severe systemic manifestations, HCV-negative cryoglobulinemia;
– polyneuropathy associated with anti-MAG antibodies;
– hematologic diseases (acute lymphoblastic leukemia, first-line or savage treatment for CD20-positive B cell non-Hodgkin’s lymphomas, first-line or savage treatment within polychemotherapy regimens for chronic lymphocytic leukemia, acute and chronic GVHD steroid-resistant, follicular lymphomas in patients not eligible for chemotherapy treatment, Hodgkin lymphoma, autoimmune hemolytic anemia, thrombotic thrombocytopenic purpura, acquired hemophilia);
– primitive or idiopathic membranous nephropathy;
– neuromyelitis optica.

Thus, the Italian NHS currently cover the use of rituximab in some neuroimmune disorders, but the use in MS is not approved and falls within the Italian Law 94/1998, by which physicians can perform off-label prescriptions (not covered by the NHS) but only in individual and exceptional cases. This represents, to date, a limit for the use in this population, due to the exceptionality and not systematicity that should characterize the prescription.

The Norwegian Institute of Public Health (NIPH) has recently conducted a cost-effectiveness evaluation of rituximab concluding that, with respect to the cladribine, rituximab generates more health in terms of QALYs and leads to a significant cost saving, while ocrelizumab, despite generating more health in terms of QALYs, induces large increases in costs (121). Moreover, the institute addressed the topic from the legal point of view too, considering whether the continued off-label use of rituximab for MS treatment could represent a legal problem when a similar preparation (ocrelizumab) is available. The discussion started from the assumption of the distinction between the right to market and the right to prescribe a medicine, underlining that the marketing authorization involves the possibility to market a drug in accordance with the terms of the authorization; however, physicians are free to prescribe a medicine, even outside these terms, if the requirements for quality, safety, and efficacy can be satisfied. Therefore, NIPH concluded that rituximab can be prescribe for MS even in the presence of ocrelizumab in the specialist health service (121, 122).

## Conclusions

DMTs demonstrated to reduce the inflammatory activity, relapse rate, and disability progression in patients with MS. However, there are still a lot of issues in terms of individual patients’ effectiveness, duration of response, safety, and compliance, which make the disease (in particular the progressive forms) an important unmet medical need.

An increasing body of evidence from RCTs and real-world studies suggest that rituximab is a highly effective DMT in relapsing MS and mildly effective in progressive MS, with low drug discontinuation rate thanks to a good safety profile and compliance. The long experience in this and other conditions, and not least a more favorable cost with respect to alternatives (especially if considering the authorized anti-CD20 ocrelizumab), highly support the use of rituximab in patients with RRMS, SPMS, or PPMS.

Most recent data have also highlighted the possibility of optimizing therapeutic scheme, with a potential further improvement of safety and efficacy and incremental saving, and suggested that rituximab may represent an optimal choice for MS women planning a pregnancy.

Currently, with the exception of the head-to-head comparison with glatiramer acetate in SPMS, no results from direct comparisons with other DMTs, including ocrelizumab, are available, but a lot of trials are ongoing, and results are awaited in the next future. However, this use could be officially recognized by national regulatory authorities, to ensure equal access for patients with MS to a therapeutic option, which demonstrated to be safe and effective not only in clinical trials (even if phase II studies but with appropriate clinical endpoints) but also in an extensive off-label use in different countries.

Finally, a dialog across Member States should began to share common standard criteria for off-label approval of medicines.

## Author Contributions

SB and LG wrote the first draft of the manuscript. FD checked and revised the draft manuscript. All authors contributed to the article and approved the submitted version.

## Funding

The open access publication fees will be funded within the AIFA pharmacovigilance grant, project ADR-648 “Monitoring of safety and efficacy of drugs prescribed under Law 648/96”.

## Conflict of Interest

The authors declare that the research was conducted in the absence of any commercial or financial relationships that could be construed as a potential conflict of interest.
