# Supplementary material for: Rituximab in Multiple Sclerosis: Are We Ready for Regulatory Approval?
Source: Front Immunol. 2021 Jul 6;12:661882. doi: 10.3389/fimmu.2021.661882 (PMC8290177; doi:10.3389/fimmu.2021.661882)
Supplement: Supplementary file 1 [file Table_1.docx]

**Supplemetary table 1:** main efficacy and safety results from clinical trials and observational studies

| **Author, year** | **Design and Population (n)** | **Treatment, dose** | **ARR** | **Relapses** | **EDSS** | **CELs** | **T2 lesions** | **Main safety findings** |
| --- | --- | --- | --- | --- | --- | --- | --- | --- |
| **Clinical Trials** | | | | | | | | |
| Bar-Or et al., 2008 | Phase I, open-label, single arm  RRMS (26) | RTX 1 g IV on week 1/3 and 24/26 | Reduction from 1.27 at baseline to 0.18 at week 72 | 80.8%of patients relapse-free. Six relapses globally reported | / | Reduction of mean number of lesions from 1.31 at baseline to 0 at week 72 | Reduction of mean number of new lesions from 0.92 at week 4 to 0 at week 72 | AEs in all patients (77% mild-moderate, 23% severe). No SAEs reported. 65.4% with infusion-related AEs, all mild-moderate; 61.5% mild–moderate infection-associated events. No PML. |
| Hauser et al., 2008 | Phase II, randomized, double-blind, placebo-controlled  RRMS (104) | RTX 1 g IV on study days 1 and 15 (n=69)  or Placebo (n=35) | Mean 0.3±0.86 RTX vs 0.8±1.20 Placebo (p=0.04) at week 24;  0.4±0.81 RTX vs 0.7±1.05 Placebo (p=0.08) at week 48 | Mean number of relapses from baseline to week 48 0.30±0.67 RTX vs 0.54±0.82 Placebo (p=0.04).  14.5% RTX patients with relapses vs 34.3% Placebo at week 24 (p=0.02); 20.3% RTX vs 40% Placebo at week 48 (p=0.04) | / | Mean number at week 24 0.5±2.0 RTX vs 5.5±1.5 Placebo (p<0.001). Mean number of new lesions at week 24 0.2±0.4 RTX vs 4.5±12.6 Placebo (p<0.001) | Reduction in lesion volume (–175 mm^3^) compared to placebo (+418 mm^3^) at week 36 (p=0.004) | More infusion-related AEs in RTX (87.3% vs 40%) after first infusion, 7.4% severe, the remaining mild–moderate. Similar numbers of SAEs (RTX 13% vs placebo 14.3%). Similar number of infections (69.6% vs 71.4%), UTI and sinusitis more common in RTX group. No PML. |
| Hawker et al., 2009 | Phase II/III randomized, double-blind, placebo-controlled  PPMS (439) | RTX 1g IV on study weeks 0, 2, 24, 26, 48, 50, 72, and 74 (n=292)  or Placebo (n=147) | / | / | Mean change from baseline to week 96 0.33±1.0 RTX vs 0.45±1.0 Placebo (p=0.34) | / | Median lesion volume change from baseline to week 96 +301.95 mm³ RTX vs +809.50 mm³ Placebo (p<0.001) | Incidence of AEs comparable between groups. 16.1% RTX vs 13.6% placebo with SAEs; 4.5% RTX vs <1% placebo serious infections. 67.1% RTX vs 23.1% placebo with infusion-related events after first infusion, decreased to placebo with successive courses. No PML. |
| Naismith et al., 2010 | Phase II, open-label, single-arm  RRMS (32) | RTX 375 mg/m^2^ IV weekly x 4 + Add-on DMT | reduction from 1.27 at baseline to 0.23 at week 52 | 57 relapses in 30 patients 18 months before study vs 7 relapses in 7 patients during the 52-week study | Improved in 7 patients, unchanged in 21 patients, and worsened in 2 patients | Reduction of mean number of new lesions per month from 2.81±0.41 to 0.33±0.1 (p<0.001) (88% reduction). Reduction of mean volume of lesions from 0.778 cm^3^, and was 0.036 cm^3^ | No significant change in mean number (27.6 pre-treatment vs 26.7 post-treatment) and mean volume (25.9 cm^3^ pre-treatment vs 24.6 cm^3^ post-treatment) | No SAEs reported. 2 patients withdrawn due to infusion reactions. 4 uncomplicated UTIs, 1 URTI. No PML. |
| de Flon et al., 2016 | Phase II, open-label, single arm  RRMS (75) | RTX 1 g IV x 2 two weeks apart | / | 1 relapse observed in 1 patient | / | Reduction of mean number of lesions per patient from 0.37±0.147 at baseline to 0.03±0.020 at 6 months (p=0.029) | Reduction of mean number of new or enlarged lesions per patient from 0.28±0.089 at baseline to 0.01±0.014 at 12 months (p=0.004) | Mild-moderate infusion reactions  as most common AEs. 3 SAEs  documented (2 cases of pyelonephritis, 1 case of influenza). No PML. |
| Honce et al., 2019 | Phase II, randomized, double-blind, placebo-controlled  RRMS (49)  CIS (4) | RTX IV on study days 1 and 15 (induction therapy) + GA 20 mg s.c. daily (n=28)  or Placebo + GA (n=27) | 0.1550 RTX-GA vs 0.3706 Placebo-GA (p=0.1219) | Patients with no relapses 74.07% RTX-GA vs 50% Placebo (p=0.07) | / | Patients with lesions 11.11% RTX-GA vs 23.08% Placebo-GA (p=0.2935). Mean number of lesions 0.11 (0.32) RTX vs 0.38 (0.80) Placebo (p=0.1081) | Patients with new lesions 25.93% RTX-GA vs 61.54% Placebo-GA (p=0.0089). Mean number of new lesions 0.48±1.22 RTX-GA vs 1.96±3.09 Placebo-GA (p=0.0270) | All participants with at least 1 AE. In RTX-GA group 18 patients with 42 infusion-associated AEs, all mild-moderate vs 11 patients with 12 infusion-AEs in Placebo-GA group. Four SAEs in RTX group, 5 in placebo group. No PML. |
| Cheshmavar et al., 2020 | Phase II/III, randomized, open-label  SPMS (73) | RTX 1 g IV every 6 months (n=37) or GA 40 mg s.c. three times per week (n=36) | Reduction from 1.30±0.52 at baseline to 0.41±0.64 at 12 months (p<0.001) RTX group vs 1.17±0.38 to 0.22±0.42 (p<0.001) GA group | / | Increase from 3.05±1.01 at baseline to 4.14±0.91 at 12 months (p<0.001) RTX group vs 3.22±1.20 to 4.60±0.67 (p<0.001) GA group | Reduction of patients with brain active lesions from 16.2% to 2.7% in RTX and from 36.1% to 8.3% in GA group. | Reduction of patients with brain active lesions from 16.2% to 2.7% in RTX and from 36.1% to 8.3% in GA group. | Eight patients (18.6%) in RTX group and 7 patients (17.1%) in GA group reported self-limited non-serious AEs. Moderate-severe allergic reactions leading to medication discontinuation reported in 4.7% (n=2) RTX patients and 2.4% (n=1) GA patients. No SAEs (as PML) observed in both groups. |
| **Observational studies** | | | | | | | | |
| Salzer et al. 2016 | Retrospective, multicenter  RRMS (557)  198 SPMS  67 PPMS | RTX 500 or 1000 mg IV every 6–12 months (in some cases after a cycle of 500 or 1000 mg twice, 2 weeks apart) | 0.044 (RRMS)  0.038 (SPMS)  0.015 (PPMS) | A total of 59 relapses reported on RTX treatment | Unchanged in RRMS (p=0.42), increased 0.5 in SPMS (p=0.10) and 1.0 in PPMS (p=0.25) | Reduction of patients with lesions from 26.2% at baseline to 4.6 post-treatment | / | Infusion-related AEs, mostly mild, in 7.8% of infusions. A total of 89 non-infusion-related AEs grade > 2 in 70 patients, mainly infections. 3 grade 2 malignancies detected, and 4 deaths (cardiac arrhythmia, respiratory failure, vascular surgery, and suicide). Only 5.2% patients discontinued RTX due to persistent disease activity or AEs. No PML. |
| Disanto et al. 2020 | Prospective, single-center  37 RRMS  22 PMS | RTX 1 g IV twice 15 days apart, then 1 g IV every 6 months, de-escalated to 500 mg IV every 6 months | / | One relapse in 1 patient under RTX 1000 mg and 0 after de-escalation to 500 mg | Stable at 12 months after de-escalation to RTX 500 mg [3.5; p=0.284] in both RRMS and PMS | No new brain and spinal lesions detected under RTX 500 mg | Only 1 new brain lesion in 1 patient and 1 new spinal lesion in 2 patients under RTX 500 mg | 33 patients (56%) reported at least one AE during the 12 months of RTX 500 mg regimen (total number of events = 52). A total of 32 infections (mainly URTI) reported in 21 patients. 3 SAEs (1 pancreatitis, 1 coronary stenting, 1 transient neutropenia). No infusion-related reactions reported. No PML. |
| Midaglia et al. 2020 | Ambispective, 2-center  303 MS | RTX 2 g IV at least during 3 cycles, followed by 1 g IV every 6 months (*high dose regimen*) or  RTX 2 g IV for the first cycle, followed by 500 mg IV every 6 months (*low dose regimen)* | 87.5% reduction in *high dose group* [from mean 0.4 to 0.05 (p<0.001)] vs 90.3% reduction in *low dose group* [from 0.31 to 0.03 (p=0.018)] at 1 year. 88.3% reduction (p=0.016) in *high dose group* vs 100% reduction (p=0.172) in *low dose group* at 3 years | / | Stable or improved in 79.4% PMS patients in *high dose group* and in 71.4% PMS patients in *low dose group* | 2.7% patients with lesions in *high dose group* vs 8% in *low dose group* at 12 months and 0% in both dose groups at 3 years | 19% patients with new lesions in *high dose group* vs 16% in *low dose group* at 1 year and 12% vs 0% at 3 years. | Higher incidence of AEs (mainly infections) in the first year in *high dose group* compared with *low dose group* (14.8% vs 4.1%). No PML. |
| Zecca et al. 2020 | Retrospective, multicenter  188 RRMS  43 PPMS  124 SPMS | RTX 375 mg/m^2^ IV twice 15 days apart or 1 g IV twice 15 days apart or 375 mg/m^2^ IV x 4 weekly; re-infusions based on CD19+ or CD27+ cells count | RRMS: from median 0.86 (95% CI: 0.73–0.99) baseline to 0.09 (95% CI: 0.07–0.13) at 2 years (p<0.0001).  SPMS: from 0.34 (0.25–0.45) to 0.06 (0.04–0.10) (p<0.0001).  PPMS: from 0.12 (0.04–0.25) to 0.07 (0.03–0.13) (p=0.45) | / | 14.6% (±0.07) RRMS patients with a 6-month confirmed EDSS progression at 3 years vs 24.7% (±0.11) SPMS and 41.5% (±0.17) PPMS  Risk of EDSS progression higher for PPMS vs RRMS patients [HR=3.28, 95% CI: 1.68–6.40, (p=0.0005)] and for SPMS vs RRMS patients [HR=2.09, 95% CI: 1.17–3.74, (p=0.013)] | 4.1% patients had new lesions at 1 year follow-up MRI. | 13.4% patients had new lesions at 1 year follow-up MRI. | At least one mild-moderate AE reported in 46.1% patients and at least one SAE in 4.4% patients. Infections (mainly respiratory and UTI) were the most common AEs (34.5%). 23.7% patients experienced at least one infusion-related reaction, mostly mild-moderate.  8 patients discontinued treatment due to AEs (mainly infections). One death occurred due to a mediastinal neoplasm. No PML. |
| Naegelin et al. 2019 | Retrospective, multicenter  88 SPMS | RTX* vs propensity matched controls never treated with RTX | / | / | Significantly lower in RTX treated patients vs patients never treated with RTX (mean difference: -0.52, 95% CI: −0.79 to −0.26; p<0.001) | / | / | AEs reported in 9% patients (n=5) [1 patient with leukocytoclastic vasculitis in both legs after first infusion, 1 patient with segmental herpes zoster infection, and 3 patients with 1 or more pneumonia events or UTI]. Two deaths reported during the follow-up period [1 due to a spontaneous intracerebral hemorrhage 3 years after stopping RTX treatment, and 1 due to pneumonia 4 years after stopping RTX treatment]. No PML. |
| Airas et al. 2020 | Retrospective, single-center  31 RRMS  16 PPMS  25 SPMS | RTX 500 or 1000 mg IV at an average interval between the first and the second dose of 7 months | / | Statistically significant reduction in relapses in RRMS and SPMS patients | RRMS: reduced in 31% patients, increased in 13%, stable in 56%.  PPMS: reduced in 18%, increased in 36%, stable in 45%.  SPMS: reduced in 20%, increased in 35%, stable in 45% | Statistically significant reduction in lesions in RRMS patients | / | 41.7% patients (n=30) experienced minor infusion-related reactions. More pronounced infusion-related  reactions only in 4% patients. One SPMS patient developed severe neutropenia and an unspecified bacterial infection after fourth RTX infusion. The most common infections during RTX treatment were lower UTIs (n=12) and URTI (n=8). No PML. |
| Dunn et al. 2018 | Cross-sectional  238 RRMS  101 PMS | RTX single IV infusions of 500 or 1000 mg 6-monthly (comparison between ADA-positive and ADA-negative treated patients) | / | 10 relapses reported in 9 patients | / | Reduction in average number of lesions in both ADA-positive (mean -0.92, 95% CI: -0.33 to 1.5) and ADA-negative patients (mean  -1.2, 95% CI: -0.61 to 1.8) [no significant differences between groups] | / | 19% patients experienced a grade 1 or 2 infusion reaction and 7% experienced a grade 1 to 3 AE. No SAEs occurred. No differences in total infusion reactions (p=0.6) and AEs (p=0.28) or severity of either were found between ADA-positive and ADA-negative patients. one ADA-negative patient discontinued due to a grade 1 AE. No PML. |
| Scotti et al. 2018 | Retrospective, single-center  43 RRMS  27 SPMS  12 PPMS  (+ 83 NTZ treated RRMS) | RTX 500 or 1000 mg IV twice 15 days apart, followed by, after 9 months, single IV infusions of 500 or 1000 mg every 6 months or NTZ | / | 3 relapses reported. No significant difference between RRMS and PMS patients (p=0.99). 2 relapses reported in NTZ treated population. | Worsening in 16.3% (n=7) RRMS vs 20.5% (n=8) PMS, with no statistically significant difference  (HR=0.87, 95% CI: 0.31-2.43, p=0.79).  EDSS worsening in 6.0% (n=5) RRMS NTZ treated patients | 1 lesion in RRMS patients vs 1 in PMS (HR=0.99, 95% CI: 0.06-15.88, p=0.99) | New lesions in 12.5% RRMS vs 2.6% PMS, with no statistically significant difference (HR=0.15, 95% CI: 0.02-1.31, p=0.09). New lesions in 7.2% RRMS NTZ treated patients. | 10 clinically relevant  infusion-associated AEs out of 339 infusions. Infections reported in 25.6% RRMS and 33.3% PMS patients. 10 patients switched from a maintenance regimen of 1000 mg/6 months to 500 mg/6 months because of recurrent infections. 6 patients (7.3%) discontinued RTX due to recurrent infections. |
| Barra et al. 2016 | Retrospective, single-center  54 RRMS  37 SPMS  6 PRMS  5 PPMS  5 unclear | RTX 1 g IV twice  2 weeks apart or single infusion of 1 g or 100 mg, followed by 500 mg x one dose, or 1 g x one dose, or 1 g x two doses | / | 29 relapses reported in 14 (25.9%) RRMS patients | Reduction from mean 2.9±1.6 at baseline to 2.5±1.8 at the end of treatment (143.9 weeks) | / | / | Mild AEs reported in 18% patients. Infusion-related reactions (none serious) reported in 8% patients. Infections (mostly UTI and URTI) reported in 36% patients. 3 patients diagnosed with a UTI required hospitalization. No malignancies observed secondary to RTX treatment. |
| D’Amico et al. 2019 | Retrospective, single-center  8 RRMS  2 SPMS  1 PPMS  2 NMO  4 NMOSD | RTX 1 g IV twice 2 weeks apart, in a single course. Administration of another course based on CD19+ | / | 14 relapses observed in 6 patients (2 RRMS, 1 SPMS, 3 NMOSD). 11 patients relapse free after RTX treatment | No EDSS worsening observed | No new lesions observed | No new lesions observed | Six AEs were recorded in 5 patients. One RRMS patient stopped RTX due to a severe lymphopaenia (<200/mm3, CTCAE 4). |
| Bellinvia et al. 2020 | Retrospective single-center  53 RRMS  12 SPMS  4 PPMS | RTX 1 g twice 8 weeks apart, followed by 1 g IV 6-monthly | global reduction from mean 0.75 at baseline to 0.36 at 12 months (p=0.004) [from 0.83 to 0.36 in RRMS; from 0.5 to 0.36 in PMS] | / | 13 patients (10 PMS and 3 RRMS) with EDSS progression at 6 months. No RRMS and 1 PMS patient with worsening at 12 months | Significant reduction of patients with MRI activity from 67.4% to 23.2% at 6 months (p<0.001) [55.4% reduction in RRMS (p<0.001) and 53.2% reduction in PMS (p<0.039)] | Significant reduction of patients with MRI activity from 67.4% to 23.2% at 6 months (p<0.001) [55.4% reduction in RRMS (p<0.001) and 53.2% reduction in PMS (p<0.039)] | Infusion-related AEs, mostly mild-moderate, reported in 43.5% patients. Non-infectious AEs, most commonly leukopenia or neutropenia (26.3%), reported in 27.5% patients. Infectious AEs, most common of which were UTI, occurred in 23.2% patients. Twelve patients (17.4%) suspended RTX, of which 4 due to AEs and 3 due to scarce tolerability. No PML. |
| Yamout et al. 2018 | Retrospective. single-center  59 RRMS  30 PMS | RTX 1 g IV twice two weeks apart, followed by single infusions of 1 g every 6–12 months | Reduction from mean 1.07±0.8 baseline to 0.11±0.26 (p<0.0001) in RRMS, and from  0.25±0.43 baseline to 0.16±0.74 (p=0.593) in PMS | A total of 15 relapses recorded. 77.9% RRMS and 90.0% PMS patients’ relapse-free | Stable/improved in 77.8% RRMS [2.89±1.62 baseline vs 2.77±2.02 (p=0.05)] and 62.5% PMS patients [5.25±1.59 baseline vs 4.91±1.96 (p=1.0)] | Proportion of patients free of new T2/Gd+ lesions: from 18.65% to 92.6% in RRMS and from 43.3% to 82% in PMS | Proportion of patients free of new T2/Gd+ lesions: from 18.65% to 92.6% in RRMS and from 43.3% to 82% in PMS | A total of 64 (71.9%) AEs recorded, mostly mild- moderate.  23 patients (25.8%) experienced a total of 40 infusion-related AEs, all mild and self-limited. 14 infections reported. 2 SAEs (pyoderma gangrenosum, increase in meningioma size) observed. No PML. |
| Alcalà et al. 2018 | Retrospective, single-center  31 RRMS  45 SPMS  14 PPMS | RTX 1 g IV twice 2 weeks apart;  another dose of  1 g when total CD19+ cells were 2% or more | 83% reduction [from 1.4 to 0.25 (p<0.001)] in RRMS and 98% reduction [from 0.55 to 0.01 (p<0.001)] in PMS. Overall 88.4% reduction [from 0.8 to 0.1 (p<0.001)] | 9 patients (8 RRMS and 1 PMS) suffered a  relapse. 90% patients remained relapse-free | Reduction from mean 4.9 to 4.6 (p=0.01) at 1 year [from 3.4 to 2.7 in RRMS and from 5.7 to 5.6 in PMS] and stable during second year of treatment | / | / | Infusion-related symptoms at first infusion, mostly mild, reported in 18.8% patients. Treatment discontinuation in 2 patients due to moderated infusion-related  reactions. Mild-moderate infections reported in 14 (16.9%) patients. No serious opportunistic infections (including PML). One patient with agranulocytosis (3 months after RTX infusion), leading to treatment discontinuation. Three patients with thrombotic events (1 died through pulmonary embolism). No newly formed neoplasms observed. |
| Hellgren et al. 2020 | Retrospective, registry-based  66 RRMS  13 SPMS  4 PPMS | RTX 1 or 2 g IV twice two weeks apart, followed by single infusions of 500 or 1000 mg IV every 6-12 months (most common 500 mg/6-monthly) | Reduction from mean 0.38±0.5 at baseline to 0.05±0.19 (p<.00001) | 8 patients (all RRMS) with relapses during RTX treatment vs 33 patients (26 RRMS and 7 PMS) in the 2 years before treatment initiation | / | One or more lesions observed in 47% patients at baseline (145 CELs/154 MRIs) vs 6% patients (18 CELs/74 MRI scans) at 1 year.  Overall reduction of CELs/MRI ratio from 0.94 to 0.24 (p<0.00001) [from 1.05 to 0.31 CELs/MRI (p=0.00003) in RRMS]. No lesions observed in PMS patients after RTX initiation | / | 48 infusion-related events recorded, mostly mild, with at least 1 reaction observed in 40 patients (48%). Four patients experienced major infusion reactions requiring an intervention. 26 patients experienced non-infusion–related AEs, most commonly infections (in 19 patients), 4 of which moderate and requiring hospitalization. Eight patients (5 RRMS and 3 PMS) discontinued RTX due to infections.  One SAE recorded (pneumonia with concomitant late-onset neutropenia). No PML. |
| Mathew et al. 2020 | Retrospective, multicenter  58 RRMS  15 SPMS  7 PPMS | RTX single dose of 500 mg as slow IV infusion every 9–12 months *(low intensity regimen)* or  RTX 500 mg twice two weeks apart followed by 500 mg every 6–12 months *(medium intensity regimen)* or  RTX 1 g twice two weeks apart followed by 1 or 2 g every 6 months eventually reduced to 500 or 1000 mg every 6 months *(high intensity regimen)* | Reduction from 0.44±0.498 baseline to 0.051±0.223 (p<0.05) at 1 year in RRMS | 97% RRMS patients had no relapses during follow-up | Improved by a score of 0.5–2.0 in 68 (85%) patients [58 RRMS, 4 SPMS, 6 PPMS], stable in 10 (12.5%) patients [9 SPMS and 1 PPMS], and worsened in 2 (2.5%) SPMS patients | No lesions (old and new) at 1 year [20% RRMS and 10% PMS patients had lesions at baseline] | / | RTX infusions well tolerated in 91% patients. Mild-moderate infusion reactions observed in 6 patients; serious infusion reaction reported in one patient.  One patient with SPMS reported recurrent UTIs during treatment. One patient with EDSS 6.0 died at home 2 weeks after RTX infusion [cause of death not known]. No severe or opportunistic infections as PML.  No neoplasms recorded.  In 3 patients became pregnant after 6 months of RTX, antepartum and post-partum periods were uneventful and newborn infants were healthy. |
| Memon et al. 2018 | Retrospective, single center  5 MS | RTX 1 g IV twice two weeks apart, followed by single infusions of 1 g every 6 or 9 months [long term continuous treatment] | / | / | / | / | / | 5 infections reported in MS population, none serious. No SAEs and no PML reported. No malignancies. |
| Vollmer et al. 2020b | Retrospective, two-center  574 RRMS  215 SPMS  118 PPMS  (77 NMOSD  16 Other) | RTX*  (mean cumulative dose of 4012 mg) | / | / | / | / | / | Neutropenia in 13 (1.5%) MS patients. Severe neutropenia (<500 cells/mm3) in 11 (1.3%) patients. Lymphopaenia in 38 (4.5%) patients. 71 (6.3%) patients hospitalized for infection. UTIs and sepsis urinary were the most common infections. No PML.  No patients had an infusion reaction considered life-threatening or resulting in hospitalization. Nine (0.9%) MS patients diagnosed with malignant cancer [mean time from RTX start to diagnosis 3.2 years]. Four (0.4%) MS patients diagnosed with a new autoimmune disease [mean time from RTX start to diagnosis 1.6 years]. Six (0.7%) MS patients diagnosed with a thromboembolic event [mean time from RTX start to diagnosis 2.6 years]. Twelve (1.3%) MS patients died within 12 months of their last RTX dose. |
| Spelman et al. 2018 | Retrospective, nationwide multicenter  1383 RRMS | RTX* (n=461) or  IFN-β/GA (n=922) [propensity score matching] | 0.003 (95% CI: 0.001-0.009) RTX vs 0.026 (95% CI: 0.020-0.033) IFN/GA (p<0.0001).  ARR reduction of 87% with RTX vs IFN/GA (HR=0.13; 95% CI: 0.03-0.56) | 3 on-treatment relapses with RTX vs 68 with IFN/GA | -0.12±0.36 with RTX vs  -0.02±0.37 with IFN/GA at 12 months (p=0.0415), and  -0.15±0.5 vs  -0.02±0.49 at 24 months (p=0.0382) | / | / | 85% reduction of drug discontinuation rate with RTX vs injectable DMTs [HR=0.15; 95% CI: 0.11-0.20]. |
| Granqvist et al. 2018 | Retrospective, multicenter  494 newly  diagnosed RRMS | RTX (n=120) 500 or 1000 mg IV every 6 months (in rare cases, the first infusion was repeated after 2 weeks) or  INF/GA (n=215) or DMF (n=86) or FTY (n=17)  or NTZ (n=50) | 0.03 RTX vs 0.21 INF/GA, 0.12 DMF, 0.16 FTY and 0.14 NTZ at follow-up | Proportion of patients with relapses: 5% (n=6) RTX vs 27% (n=58) INF/GA, 11.6% (n=10) DMF, 17.6% (n=3) FTY, 20% (n=10) NTZ | / | Proportion of patients with lesions: 1.7% RTX vs 12.6% INF/GA (p<0.01),  12.8% DMF (p=0.05), 5.9% FTY (p=0.46), and 6% NTZ (p=0.07) | / | Mild AEs more common for INF/GA- (60.9% patients) and DMF- (75.6%) compared to RTX- (24.2%) treated patients; moderate-severe AEs equally low. AEs did not differ significantly between RTX, FTY, and NTZ. No SAEs reported in RTX group. The proportion of patients discontinuing treatment was significantly higher with INF/GA (80.5%), DMF (37.2%), FTY (47.1%) and NTZ (48%) compared with RTX (5.8%). |
| Boremalm et al. 2019 | Retrospective, multicenter, registry-based  241 RRMS patients switched from first-line INF/GA | RTX (n=48) 500 or 1000 mg IV every 6 months or NTZ (n=105) or FTY (n=88) | 0.03 RTX vs 0.02 NTZ vs 0.07 FTY | 6 (5.7%) patients with relapses in NTZ group vs 4 (8.3%) in RTX [adjusted HR: 1.0, 95% CI 0.2–5.6] vs 16 (18.2%) in FTY [adjusted HR: 3.4, 95% CI 1.3–9.2] | / | patients with ≥ 1 MRI scan with CEL(s): 1 RTX (2.1%); 1 NTZ (1.0%); 11 FTY (12.5%) | / | Patients with at least 1 AE: 5 (10.4%) in RTX, 8 (7.6%) in NTZ, and 6 (6.8%) in FTY group. Incidence of AEs per year: 0.04 for RTX-treated, 0.03 for NTZ-treated, and 0.03 for FTY-treated patients. Five grade 2 (cough, depression, genital herpes, kidney infection, and pneumonia) and 1 grade 5 event (a suicide due to overdosing of sedatives with a severe concomitant psychiatric illness) in RTX group vs 4 grade 2, 3 grade 3 and 1 life-threatening grade 4 (depression) events in NTZ group and 4 grade 2 and 3 grade 3 events in FTY group.  No severe infusion-related AEs reported.  Only 1 RTX-treated patient (2.1%) discontinued treatment due to pregnancy vs 47 (44.8%) NTZ-treated and 35 (39.8%) FTY-treated patients. |
| Vollmer et al. 2020a | Retrospective, single-center  1004 RRMS  176 SPMS  66 PPMS | RTX (n=182) 1 g IV twice two weeks apart, followed by single infusions of 500 mg every 6 months or  NTZ (n=451) or  FTY (n=271) or  DMF (n=342) | / | Percentage of patients experiencing clinical relapses: 3.3% RTX vs 6.0% NTZ (p=0.060), 8.9% FTY (p=0.021), 12.9% DMF (p<0.001). | / | Percentage of patients with lesions: 0.7% RTX vs 5.8% NTZ (p=0.010), 13.1% FTY (p<0.001), 10.0% DMF (p<0.001). | Percentage of patients with new lesions: 17.6% RTX vs 25.3 NTZ (p=0.058), 35.0% FTY (p<0.001), 31.5% DMF (p=0.003). | No significant difference observed for NTZ vs RTX, while FTY- and DMF-treated patients had greater OR of discontinuation [FTY: OR=2.02, 95% CI: 1.24–3.30, p=0.005; DMF: OR=3.27, 95% CI: 2.15–4.97, (p<0.001)].  Infections are the most common AEs leading to discontinuation of RTX (2.7% of all RTX-treated patients). For NTZ, the most common AEs leading to discontinuation were flushing, rashes, or hot flashes (3.1%), while for both FTY and DMF patients were GI-related issues (FTY: 4.1%; DMF: 19.3%) |
| Evertsson et al. 2020 | Retrospective, two-centers  386 RRMS  86 SPMS | RTX (n=311) single infusion of 500 or 1000 mg IV, followed by single infusions of 500 mg every 5–7 months or  OCR (n=161)  300 mg IV twice two weeks apart, followed by single infusions  of 600 mg every 5–7 months | / | / | / | / | / | Infections were the most common AEs in OCR group (n=10), while no infections were reported in RTX group. Infusion-related reaction reported in 2 OCR- and 2 RTX-treated patients. 10% patients with RTX vs 15% with OCR discontinued treatment [no statistically significant difference, p=0.11]. Discontinuation due to AEs was more common with OCR than with RTX (9.3% vs 2.6%, p<0.01). 2.5% OCR vs 1.6% RTX discontinued treatment due to lack of effect. |
| Perez et al. 2020 | Prospective, single-center  96 RRMS  41 SPMS  8 PPMS | MabThera® (n=105) or  Truxima® (n=40)  1 g IV twice 2 weeks apart, followed by a single infusion of 1 g | Reduction from 0.50 to 0.02 with MabThera® vs 0.40 to 0.025 with Truxima® (p=1) | 3 relapses reported on-treatment (1 MabThera® vs 2 Truxima®) | / | Percentage of patients with lesions at first MRI after RTX start: from 20% to 1% MabThera® vs from 21% to 0% Truxima® (p=0.41).  No lesions on the second MRI. | Percentage of patients with new lesions at first MRI after RTX start: from 38% to 10% MabThera® vs from 35% to 12% Truxima® (p=0.76).  Only 1 MabThera® patient with a new lesion on the second MRI. | Mild-moderate infusion-related reactions were the most frequent AEs in both groups. No patients had serious reactions and no infusion incomplete. The most common non infusion-related AEs were UTIs [16 patients (15.5%) with MabThera® and 4 (10%) with Truxima®] and low-grade asthenia [7 patients (6.7%) and 6 (15%), respectively]. No severe infections and no PML reported. No patients discontinued RTX after 1 year. |
| Alcalà et al. 2019 | Retrospective, two-centers  55 RRMS switched from FTY | RTX (n=27) 1 g IV twice 15 days apart, followed by a single infusion of 1 g when total CD19+ cells were 2% or more  ALT (n=28) | Reduction from 1.24 baseline to 0.02 (p<0.001) RTX vs from 1.29 to 0.004 (p<0.001) ALT | Patients free from relapses: 22 (81.5%) RTX vs 26 (92.9%) ALT (p=0.21) | reduction from median 3.5 (2–4) baseline to 2.5 (2–4) at 12 months (p<0.01) RTX vs from 2.8 (2–3) to 2.0 (1.5–2.5) (p=0.03) ALT | Patients free from radiological activity: 21 (80.8%) RTX vs 24 (85.7%) ALT (p=0.63) | Patients free from radiological activity: 21 (80.8%) RTX vs  24 (85.7%) ALT (p=0.63) | Infusion-related reactions (mostly mild) most frequent AEs in both groups: in 16 ALT-treated (57.1%) and in 7 (29.2%) RTX-treated patients. Fourteen (51.9%) ALT-treated patients and 5 (18.5%) RTX-treated patients had common infections (respiratory and urinary) with good recovery with standard treatment. No severe or opportunistic infections reported.  One patient treated with RTX + oral contraceptives suffered a DVT (complete recovery); another RTX-treated patient was diagnosed with breast neoplasm 3 months after infusion. RTX was discontinued in 6 patients (vs 1 ALT) due to suboptimal response (2 patients), safety concerns (3 patients - serum sickness, persistent pruritus, diagnosis of breast cancer), or concomitant conditions (1 patient - aggressive psoriasis). |
| Berenguer-Ruiz et al 2016 | Prospective,  single-center  12 RRMS | RTX 375 mg/m^2^ IV every 4 weeks or 1 g IV twice 2 weeks apart  [as second/third-line treatment] | / | No patients experienced a  clinical relapse  [12 patients (100%) relapse-free] | Improved in 11 patients and stable in 1 patient [mean 3.75±1.67 at baseline vs 2.21±1.3 at the end of observation period] | No MRI activity detected | No MRI activity detected | No patients experienced SAEs or discontinued treatment. Four patients suffered mild infusion-related AEs (headache, flushing, and pruritus). Infection-associated events (URTIs and UTIs), all mild, reported in 7 patients. |
| Durozard et al. 2019 | Nationwide retrospective multicenter  50 RRMS | RTX 375 mg/m^2^ IV x 4 weekly or 1 g twice two weeks apart, followed by single infusions of 1 g every 6 months, or 1 g IV twice two weeks apart every 6 months  [as second/third-line treatment] | Mean 0.18 during RTX treatment vs 0.8 during last DMT (p<0.0001),  vs 1 at onset (p<0.0001), and vs 1.6 in the year before RTX initiation (p<0.0001) | A total of 12 relapses reported in 10 patients after RTX initiation | Reduction from median 4.6 (0-7) at RTX initiation to 4.0 (0-7.5) at last clinical evaluation (p<0.0001).  Decreased in 23 patients (median = –1; range -0.5 to -2.5), unchanged in 25 patients, and increased in 2 patients (0.5 and 1 point). | Percentage of patients with lesions: 72% at baseline vs 8% at first MRI after RTX initiation (p<0.0001) | / | Around 16 AEs reported, and 10 patients with at least one AE (mainly infections). Three infusion-related reactions observed. Three grade 3 AEs recorded. No deaths and no PML.  Two patients discontinued treatment (one due to an AE and one due to pregnancy). |
| Malucchi et al. 2015 | Case report  10 RRMS switched from NTZ | RTX 375 mg/m^2^ once/week for 4 weeks, followed by 1 g twice two weeks apart in case of CD19+ increase and/or clinical or radiological disease reactivation | / | / | Unchanged at 6 and 12 months | No lesions at 6 months | No new lesions at 6 months | / |
| Lo Re et al. 2015 [225] | Retrospective, two-centers  132 RRMS who interrupted NTZ | RTX* (n=7) or  FTY (n=57) or  IFN/GA/ teriflunomide/  AZA (n=16) or  NTZ (n=9) or  cyclophosphami-de or mitoxantrone (n=4) or  AHSCT (n=2) or  treatment free (n=37) | No evidence of disease clinical and radiological reactivation observed in RTX cohort | No evidence of disease clinical and radiological reactivation observed in RTX cohort | No evidence of disease clinical and radiological reactivation observed in RTX cohort | No evidence of disease clinical and radiological reactivation observed in RTX cohort | No evidence of disease clinical and radiological reactivation observed in RTX cohort | / |
| Alping et al. 2016 | Retrospective, multicenter, registry-based  256 RRMS who suspended NTZ because of PML risk | RTX (n=114) 500 or 1000 mg IV every 6 months; in some cases, the first infusion had been repeated after 2 weeks or  FTY (n=142) | 0.02 in RTX vs 0.16 in FTY group | Percentage of patients with relapses in the first 1.5 years of treatment: 1.8% in RTX vs 17.6 in FTY group [adjusted HR: 0.09, 95% CI 0.02–0.40] | / | Percentage of patients with  lesions in the first 1.5 years of treatment: 1% in RTX vs 16% in FTY group [OR: 0.05, 95% CI 0.00–0.22] | Percentage of patients with Gd+/new T2 lesions: 0.8% in RTX vs 22% in FTY group | More AEs reported in FTY group (21%) compared to RTX group (5%). One grade 3, 1 grade 2 and 4 grade 1 events reported in RTX group. In the FTY cohort, one severe, potentially life-threatening grade 4 event [laryngeal edema occurring in the setting of a bacterial infection, necessitating invasive ventilation], 2 grade 3, 8 grade 2, and 28 grade 1 events reported. Reactions related to first infusion recorded in 26% patients in RTX group (all grade 1), vs 7% patients in FTY group. Less drug discontinuation reported in RTX (1.8%) vs FTY group (28.2%) |
| Razaz et al. 2020 | Retrospective, registry-based  668 RRMS  32 SPMS  8 PPMS  [pregnant women] | Suspended RTX* (n=76) or  suspended NTZ (n=53) or  untreated (n=457) | Pre-pregnancy: 0.05-0.04. During pregnancy: 0.01 RTX, 0.15 NTZ, 0.13 untreated. One year post-partum: 0.05 RTX, 0.40 NTZ, 0.23 untreated.  Adjusted RR 1 year post-partum NTZ vs RTX: 7.65 (95% CI: 2.47–23.6); untreated vs RTX: 4.69 (95% CI: 1.67–13.2) | / | / | Patients with lesions within 12 months post-partum: 1 (1.3%) in RTX group, 6 (11.3%) in NTZ group, 45 (9.8%) in untreated cohort | / | / |
| Kumpfel et al. 2021 | Retrospective  64 MS  (17 NMOSD) | RTX* (n=25) or OCR (n=39) | / | All MS patients in both RTX and OCR cohort were  relapse free  during pregnancy. Five RRMS patients experienced a relapse postpartum | stable during pregnancy in all patients | / | / | Significantly more preterm births (9.8% vs 45%) occurred after exposure to RTX or OCR during pregnancy. Two major congenital abnormalities reported (3.3%) were observed. Severe infections during pregnancies occurred in 3 mothers and infections leading to hospitalization in 3 newborns. |
| Das et al. 2018 | Retrospective, single-center, case series  7 MS  (3 NMOSD) | RTX* | / | only 1 patient with NMOSD relapsed post-partum. | / | / | / | 9/10 pregnancies resulted in term live births, with healthy children. Medical complications, including both pregnancy-related (gestational diabetes, pre-eclampsia) and disease-related (relapses), reported in 4/10 pregnancies. |
| Canibaño et al. 2020 | Case Report  1 RRMS pregnant woman discontinuing FTY | RTX two 1 g IV infusions 15 days apart; 1 g six-monthly post-delivery | / | No relapses recorded both during pregnancy and post-delivery | Improvement from 7.0 to 4.5 during RTX and 4.0 post-delivery | / | / | No pre-term delivery.  Newborn APGAR score 9 at 1 min, and 10 at 5 min. No infant infections at 3 months, and normal development. Infant's %  CD19+ count 19.8%.  No neurologic or infectious sequelae reported in the mother at 6-month follow-up. |

* no dose regimens reported.

AE = adverse event; AHSCT = autologous haematopoietic stem cell transplantation; ALT: alemtuzumab; AZA = azathioprine; CELs: contrast-enhancing lesions; CI = confidence interval; CIS =clinically isolated syndrome; DMF = dimethyl fumarate; DMTs: disease modifying therapies; FTY = fingolimod; GA = glatiramer acetate; Gd+ = gadolinium-enhancing; HR = hazard ratio; INF = interferon; IV = intravenous; MRI = Magnetic Resonance Imaging; NMOSD = neuromyelitis optica spectrum disorder; NTZ = natalizumab; OCR = ocrelizumab; PML = progressive multifocal leukoencephalopathy; PMS = progressive multiple sclerosis; PPMS = primary progressive multiple sclerosis; PRMS: Progressive-relapsing multiple sclerosis; RRMS = relapsing remitting multiple sclerosis; RTX = rituximab; SAE = serious adverse event; SPMS = secondary progressive multiple sclerosis; URTI: upper respiratory tract infection; UTI: urinary tract infection
